# Supplementary material for: Atomic-scale insights into surface reconstruction and transformation in Co-Cr spinel oxides during the oxygen evolution reaction
Source: Nat Commun. 2025 Nov 10;16:9895. doi: 10.1038/s41467-025-65626-x (PMC12603048; doi:10.1038/s41467-025-65626-x)
Supplement: Supplementary file 1 — Supplementary file [file 41467_2025_65626_MOESM1_ESM.pdf]

Supplementary Information of

Atomic-scale insights into surface reconstruction and transformation in Co-Cr spinel oxides during the oxygen evolution reaction

Biao He[1], Pouya Hosseini[1,2], Tatiana Priamushko[3], Oliver Trost[4], Eko Budiyanto[5], Christoph Bondue[4], Jonas Schulwitz[6], Aleksander Kostka[7], Harun Tüysüz[5,8], Martin Muhler[6], Serhiy Cherevko[3], Kristina Tschulik[2,4], Tong Li[1]*

1 Faculty of Mechanical Engineering, Atomic-scale Characterisation, Ruhr-Universität Bochum, Universitätsstraße 150, 44801 Bochum, Germany

2 Max-Planck-Institut für Nachhaltige Materialien GmbH, Max-Planck-Straße 1, 40237 Düsseldorf, Germany

3 Forschungszentrum Jülich GmbH, Helmholtz-Institute Erlangen-Nürnberg for Renewable Energy (IET-2), 91058 Erlangen , Germany

4 Faculty of Chemistry and Biochemistry, Analytical Chemistry II, Ruhr-Universität Bochum, Universitätsstraße 150, 44801 Bochum, Germany

5 Department of Heterogeneous Catalysis, Max-Planck-Institut für Kohlenforschung, Kaiser-Wilhelm-Platz 1, 45470 Mülheim an der Ruhr, Germany

6 Faculty of Chemistry and Biochemistry, Laboratory of Industrial Chemistry, Ruhr-Universität Bochum, Universitätsstraße 150, 44801 Bochum, Germany

7 Zentrum für Grenzflächendominierte Höchstleistungswerkstoffe (ZGH), Ruhr-Universität Bochum, Universitätsstraße 150, 44801 Bochum, Germany

8 Catalysis and Energy Materials Group, IMDEA Materials Institute, Calle Eric Kandel 2, 28906 Getafe, Madrid, Spain

***Corresponding author**

E-mail: tong.li@rub.de

Tel: +49 (0)234 32 26099

Postal address: Universitätsstr.150, Bochum, 44801, Germany

Supplementary Figures

Figure S1 Structure and morphology characterization of pristine nanoparticles 3
Figure S2 Linear sweep voltammetry (LSV) curves of Co_2CrO_4 and $CoCr_2O_4$ 4
Figure S3 LSV plots of pristine states normalized by electrochemical active surface area and chronopotentiometry measurements 5
Figure S4 Tafel slope for Co_2CrO_4 and $CoCr_2O_4$ 6
Figure S5 Charge capacity and oxygen intercalation comparison 7
Figure S6 Electrochemical double-layer capacity and ECSA 8
Figure S7 Cyclic voltammetry profiles of $CoCr_2O_4$ with a limited potential window 9
Figure S8 Co oxidation state derived from X-ray absorption near-edge structure data 10
Figure S9 Electrochemical Impedance Spectroscopy of $CoCr_2O_4$ and Co_2CrO_4 11
Figure S10 Raman spectra of reference samples 12
Figure S11 Selected area electron diffraction (SAED) of $CoCr_2O_4$ before and after oxygen evolution reaction (OER) 13
Figure S12 SAED pattern of Co_2CrO_4 before and after OER 14
Figure S13 Mass spectrum of atom probe tomography (APT) data of $CoCr_2O_4$ nanoparticles 15
Figure S14 APT data of $CoCr_2O_4$ nanoparticles before and after OER 16
Figure S15 Additional APT data of $CoCr_2O_4$ before and after OER 17
Figure S16 Additional high resolution transmission electron microscopy (HRTEM) pictures of $CoCr_2O_4$ after OER 18
Figure S17 APT data of $CoCr_2O_4$ and Co_2CrO_4 after OER showing K distribution 19
Figure S18 Local mass spectra from different regions of APT data of $CoCr_2O_4$ 20
Figure S19 TEM image of 1000-cycle $CoCr_2O_4$ showing particle size distribution after OER 21
Figure S20 APT data of Co_2CrO_4 nanoparticles before and after OER 22
Figure S21 Additional APT reconstruction of Co_2CrO_4 23
Figure S22 3D-APT reconstruction of non-segregated-Co_2CrO_4 nanoparticles 24
Figure S23 Additional APT reconstruction of non-segregated-Co_2CrO_4 nanoparticles 25
Figure S24 Additional HRTEM pictures of Co_2CrO_4 26
Figure S25 TEM image of 1000-cycle $CoCr_2O_4$ size distribution after OER 27
Figure S26 pH dependence electrochemical measurements of $CoCr_2O_4$ and Co_2CrO_4 28
Figure S27 Differential Electrochemical Mass Spectrometry (DEMS) on Co_2CrO_4 29
Figure S28 DEMS measurements on $CoCr_2O_4$. 30
Figure S29 $\chi(^{16}O^{18}O)$ changes with cycles in DEMS data 31
Figure S30 Schematic figure of the DEMS cell 32

Supplementary Tables

Supplementary Table 1. Cr, Co and O concentrations from TEM/EDS 33
Supplementary Table 2. EIS simulation results 33
Supplementary Table 3. Fitting results in the SAED pattern of $CoCr_2O_4$ 33
Supplementary Table 3. Fitting results in the SAED pattern of Co_2CrO_4 34

Supplementary Notes

Supplementary Note 1 S number calculation 35
Supplementary Note 2 XPS measurements 36
Supplementary Note 3 OD counts from APT data 37
Supplementary Note 4 DEMS measurements 38-39

Supplementary References 39

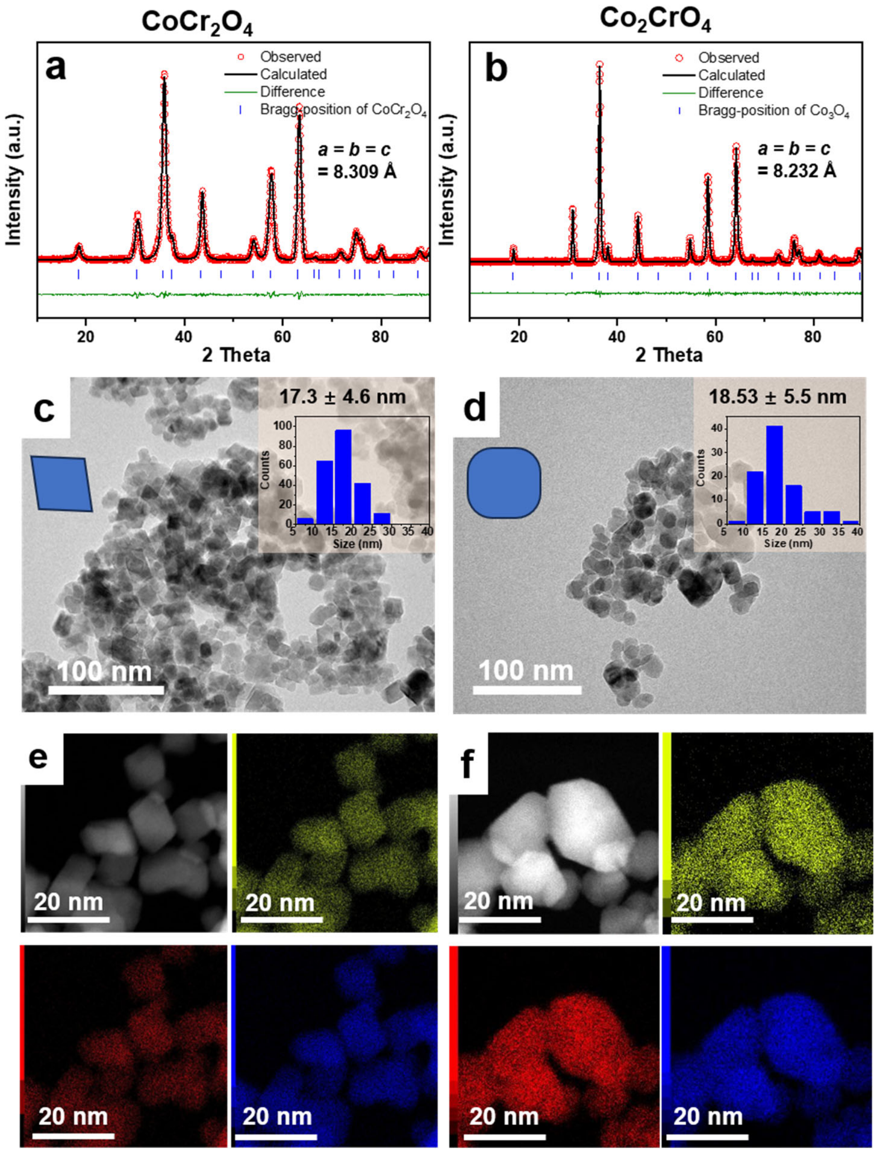

Figure S1. Structure and morphology characterization of pristine nanoparticles. Powder X-ray diffraction (XRD) of the pristine (a) $CoCr_2O_4$ and (b) Co_2CrO_4 nanoparticles. The Rietveld XRD analysis shows that both oxides fit well with the spinel structure using JCPDS card No. 80-1540 for $CoCr_2O_4$ refinement and JCPDS card No. 80-1668 for Co_2CrO_4 refinement. The low-resolution transmission electron microscopy (TEM) images of (c) $CoCr_2O_4$ and (d) Co_2CrO_4 show a homogeneous dispersion of nanoparticles with the size distribution histogram in the insets (errors bars are obtained from standard deviation), and the corresponding blue features in the TEM image represent the most visible shape in the total number of nanoparticles; Energy dispersive spectrometer (EDS) mapping of (e) $CoCr_2O_4$ and (f) Co_2CrO_4 in the pristine state. In EDS/TEM measurement, a seemingly homogeneous distribution of Co, Cr, and O elements is observed.

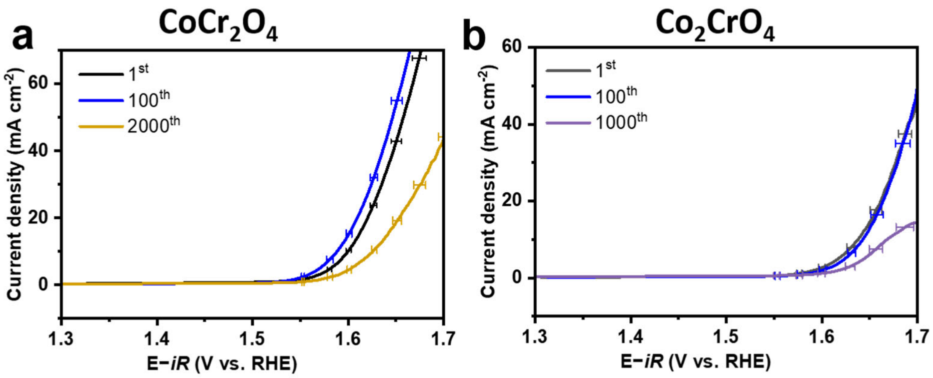

Figure S2. Linear sweep voltammetry (LSV) curves after the first, 100th, and 1000th/2000th cyclic voltammetry (CV) cycles with error bars obtained from the three measurements. The LSV measurements are conducted in 1 M KOH with a pH value of 14.00±0.01, and on 0.196 cm^2 glassy carbon electrode with a mass loading of ~0.05 mg at room temperature. The scan rate is 10 mV dec^{-1} within the potential range of 1-1.7 V vs. RHE, and rotation speed is 1600 rpm. The compensation resistances (90%) are done automatically by the potentiostat.

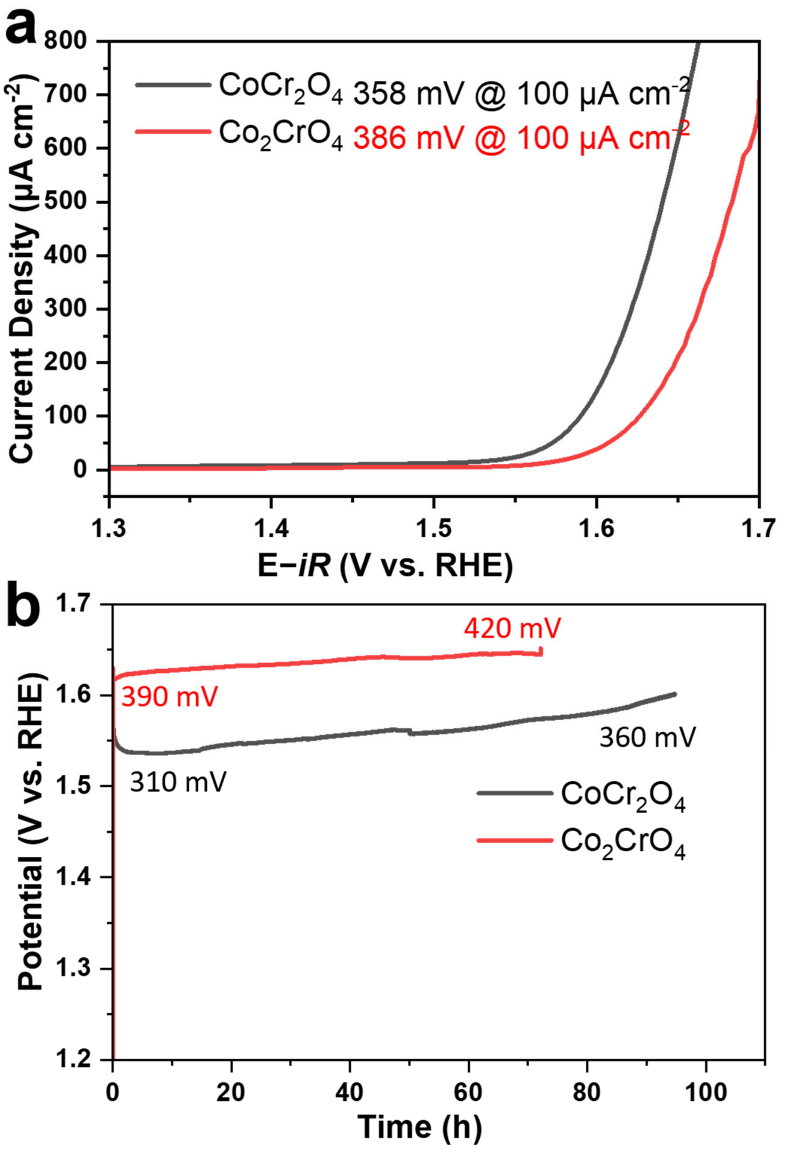

Figure S3. (a) LSV plots of pristine states for both samples that are normalized by electrochemical active surface area (ECSA) with corresponding overpotential at 100 µA cm^{-2}. (b) Long-term stabilities of both $CoCr_2O_4$ and Co_2CrO_4. The chronopotentiometry (CP) measurements were employed at a constant current density of 10 mA cm^{-2} in 1 M KOH with carbon paper as the working electrode and the loading area is around 0.2 cm without *iR* compensation. Before the measurement, 50 CV cycles were performed to allow the electrolyte penetration and activate the electrode materials.

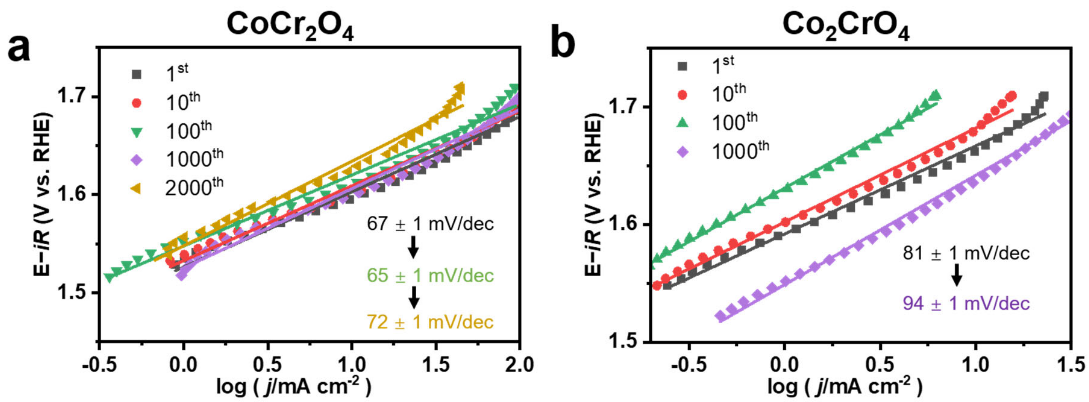

Figure S4. Tafel slope of (a) $CoCr_2O_4$ and (b) Co_2CrO_4 nanoparticles that are obtained from LSV data at different stages, and the errors are obtained from the fitting. For $CoCr_2O_4$: The Tafel slope decreases slightly from 67 ± 1 to 65 ± 1 mV/dec after 100 cycles, indicating improved OER kinetics due to formation of conductive, active (Co,Cr)-based (oxy)hydroxides, which is in line with most Co-based catalyst with a Tafel plot of around 60 mV/dec. The slope remains relatively stable afterward, consistent with the long-term durability as 72 ± 1 mV/dec after 2000 cycles was obtained. For Co_2CrO_4, the Tafel slope remains high and increases slightly with cycling, from 81 ± 1 mV/dec in pristine to 94 ± 1 mV/dec after 1000 cycles, reflecting a lack of kinetic improvement and suggesting a degradation during long cycles.

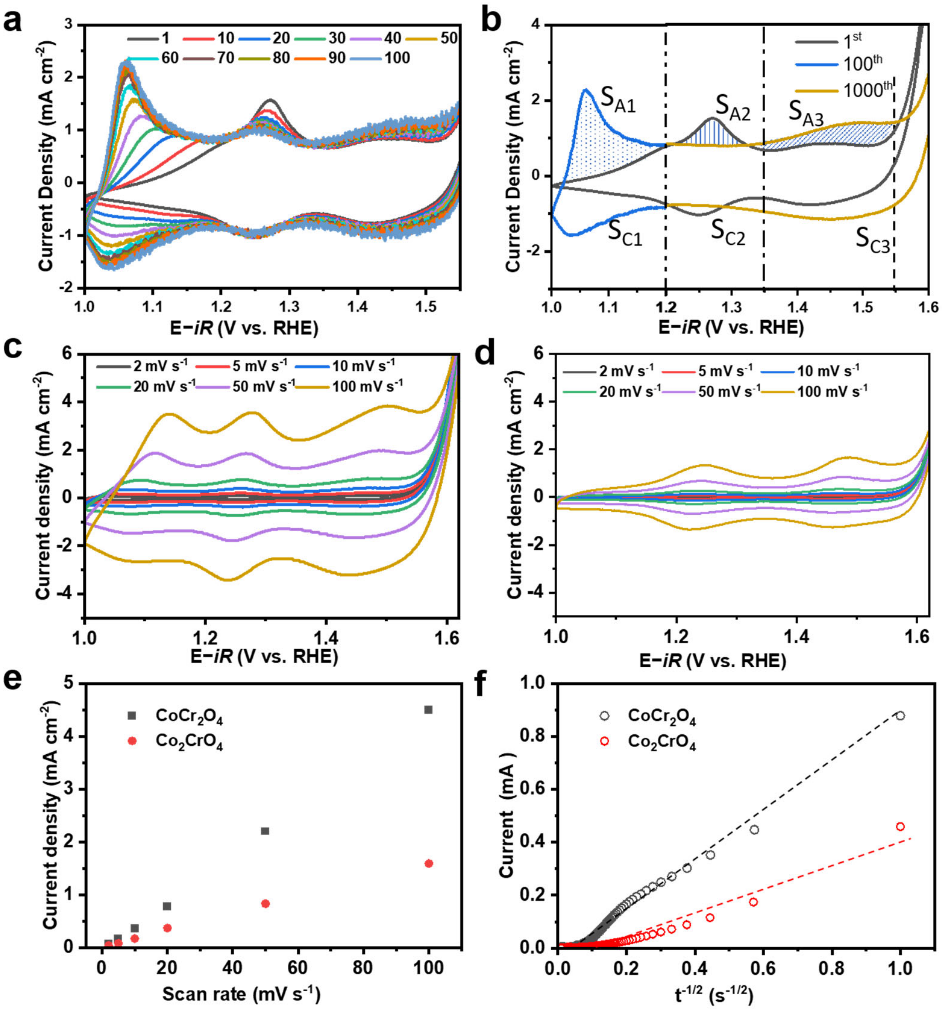

Figure S5. (a) CV data of $CoCr_2O_4$ sample before 100 cycles showing the redox peaks evolution. (b) The 1st, 100th, and 100th CV profile is presented as the representative to calculate the integral region differences that correspond to A1/C1, A2/C2, and A3/C3 peaks in terms of areas S_{A1}/S_{C1}, S_{A2}/S_{C2}, and S_{A3}/S_{C3}, respectively. For each region, the cycle with the lowest current density is selected as the baseline, which is 1st cycle for S_{A1}/S_{C1} and S_{A3}/S_{C3}, and the 2000th cycle for S_{A2}/S_{C2}. The charge density values difference, ΔQ (Q_A - Q_C), listed in Table 1, are derived by Q = S/scan rate. As these charge densities include contributions from various sources, such as capacitive, classical Faradaic, and pseudocapacitive, the charge difference was utilized in the charge balance calculations to reduce the impact of these overlapping contributions and isolate the relevant data. CV curves of (c) $CoCr_2O_4$ and (d) Co_2CrO_4 at various scan rates from 2 mV s^{-1} to 100 mV s^{-1}, where redox peaks indicate the electrochemical oxygen intercalation/de-intercalation. (e) The current density comparison of first anodic peak (A1) at each scan rate. (f) The chronoamperometry data (i vs. $t^{-1/2}$) used for the calculation of oxygen ion diffusion coefficients. All CV measurements are conducted in 1 M KOH with a pH value of 14.00±0.01, and on 0.196 cm^2 glassy carbon electrode with a mass loading of ~0.05 mg at room temperature. The potential range used is 1-1.65 V vs. RHE, and rotation speed is 1600 rpm. The compensation resistances are done automatically by the potentiostat.

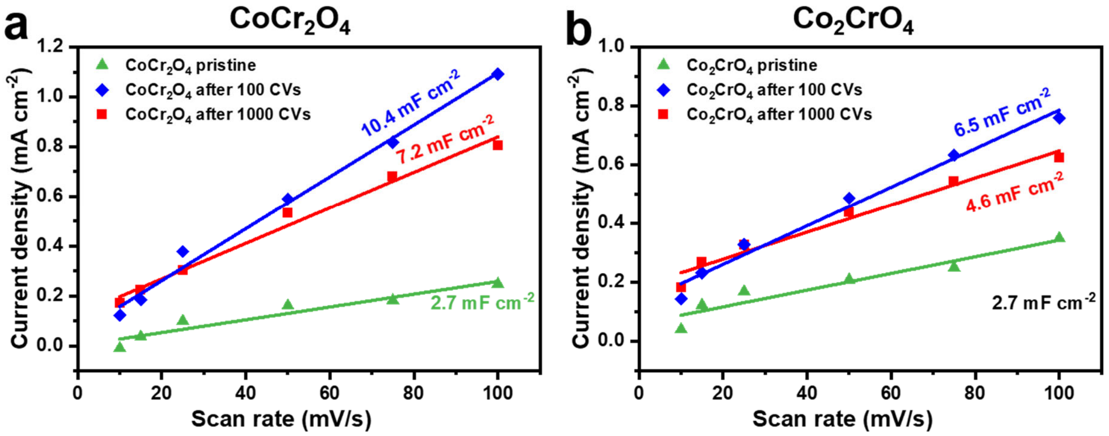

Figure S6. Electrochemical double-layer capacity (C_{dl}) in pristine, after 100 and after 1000 cycles for (a) $CoCr_2O_4$ and (b) Co_2CrO_4 nanoparticles that are obtained from CV. All CV measurements are conducted in 1 M KOH with a pH value of 14.00±0.01, and on 0.196 cm^2 glassy carbon electrode with a mass loading of ~0.05 mg at room temperature. CV curves were obtained from 0.80 to 0.90 V vs. RHE, where there was no Faradaic current, at scan rate of 10, 15, 25, 50, 75 and 100 mV dec^{-1}, and rotation speed is 1600 rpm. The compensation resistances are done automatically by the potentiostat. After 100 CV cycles, the $CoCr_2O_4$ nanoparticle shows an increase in the electrochemical surface area (ECSA), while its value is slightly decreased after 1000 cycles. For the Co_2CrO_4 nanoparticle, the ECSA also increased before 100 cycles and then decreased after 1000 cycles.

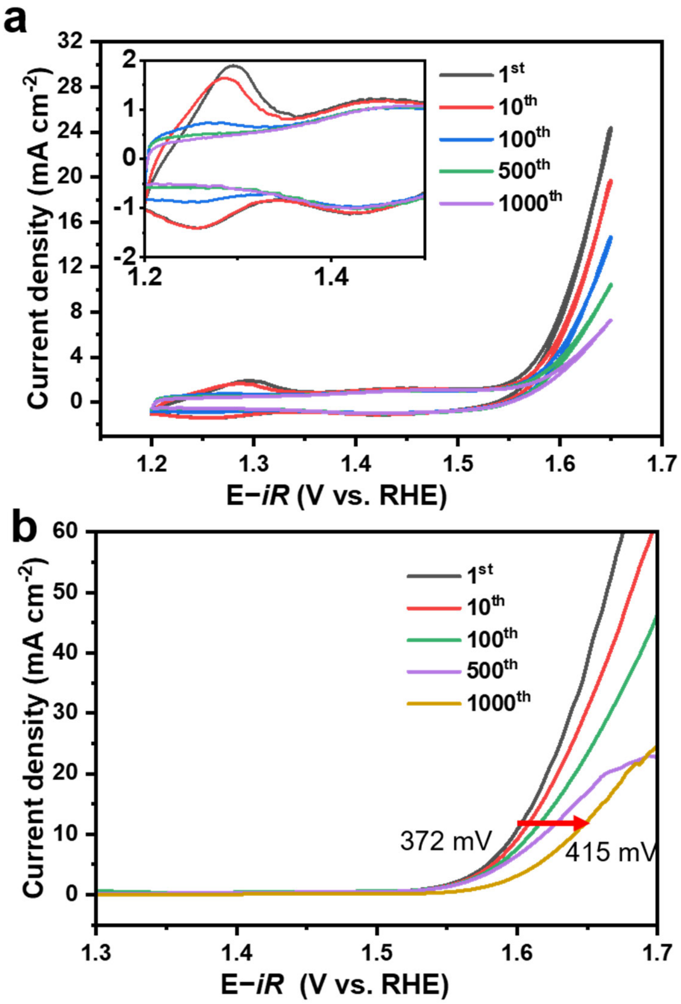

Figure S7. (a) CV profiles recorded at a scan rate of 50 mV/s of $CoCr_2O_4$ in pristine, 10, 100, 500, and 1000 cycles indicated in the figure with a potential limit of 1.2-1.7 V vs. RHE. (b) Corresponding LSV profiles recorded at a scan rate of 10 mV/s of $CoCr_2O_4$ after 1, 10, 100, 500, and 1000 CV cycles with a potential limitation of 1.2-1.7 V vs. RHE. These measurements are conducted in 1 M KOH with a pH value of 14.00±0.01, and on 0.196 cm^2 glassy carbon electrode with a mass loading of ~0.05 mg at room temperature. The rotation speed is 1600 rpm, and the compensation resistances are done automatically by the potentiostat.

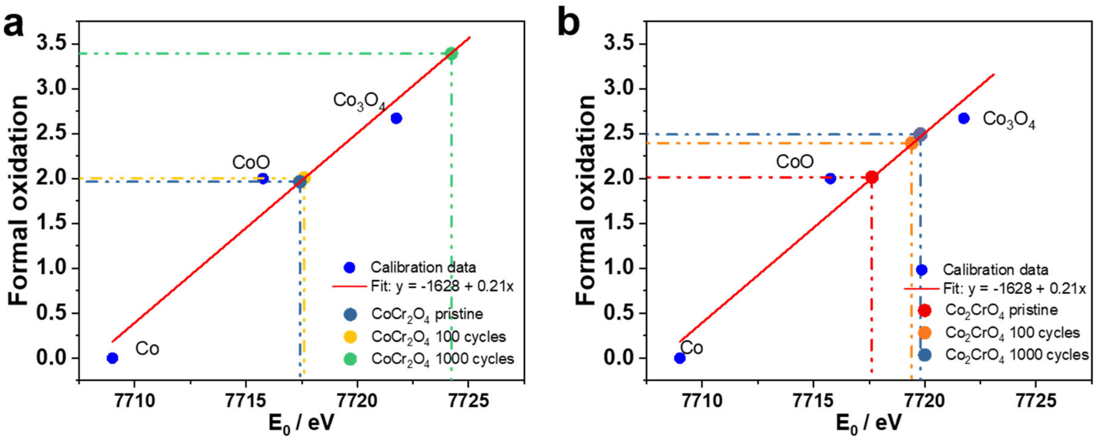

Figure S8. The oxidation state estimation of Co in (a) $CoCr_2O_4$ and (b) Co_2CrO_4 by edge position fitting of the K-edge X-ray absorption near-edge structure (XANES) spectroscopy. The blue dots indicate the position of the first inflection point (obtained from the first derivative) of the Co edge for calibration.

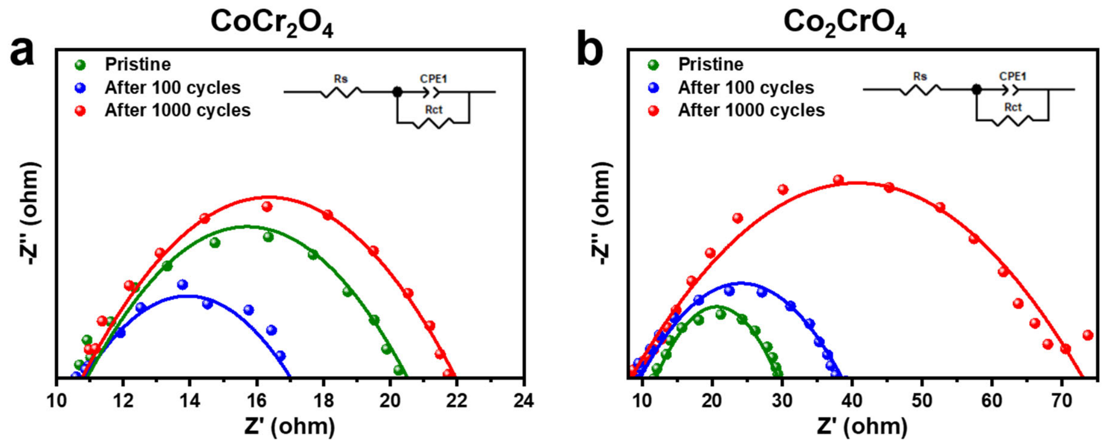

Figure S9. Electrochemical impedance spectroscopy (EIS) data of (a) $CoCr_2O_4$ and (b) Co_2CrO_4 during OER at 1.60 V vs. RHE. The equivalent circuit model for $CoCr_2O_4$ in series includes an ohmic resistor, which is predominantly defined by the electrolyte resistance between the working and reference electrodes. This setup is considered an electric double-layer capacitor connected in parallel to the faradaic resistance of the oxygen evolution reaction (OER) as indicated in the insets. Despite the OER mechanism involving multiple electron transfer stages, the associated serial resistances are parallel to the same capacitor, resulting in only one semicircle. The EIS simulation results obtained by Zviwer software are presented in Supplementary Table 2.

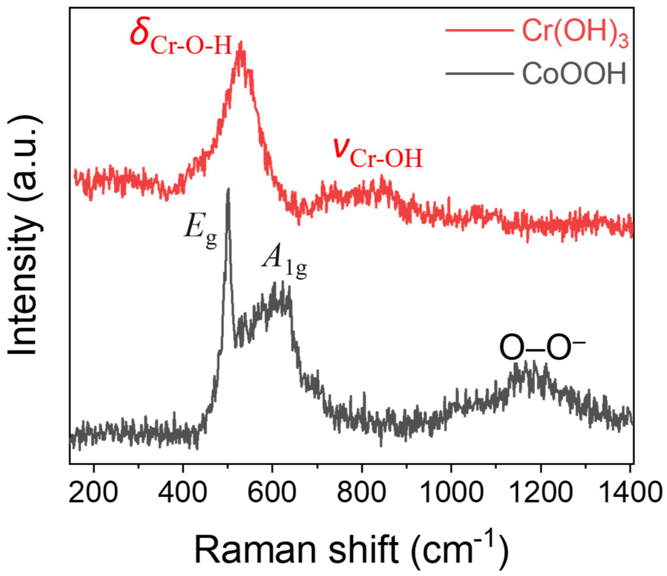

Figure S10. Raman spectra of as-synthesized reference $Cr(OH)_3$ and CoOOH materials.

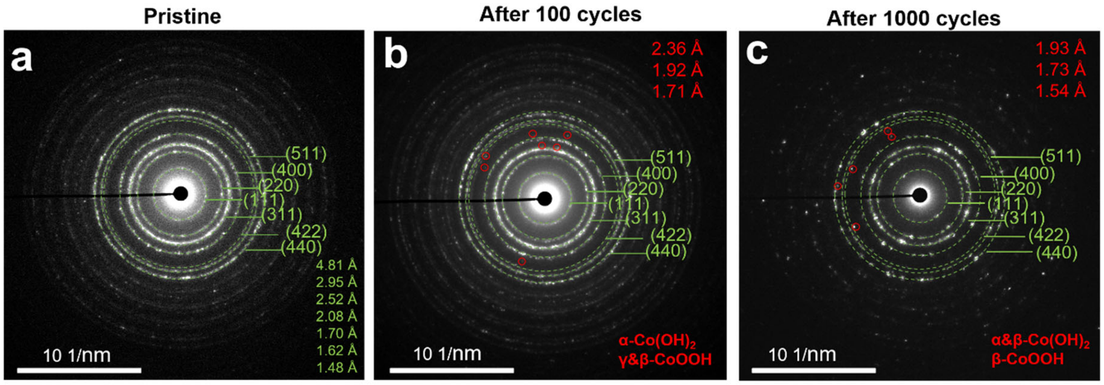

Figure S11. Selected area electron diffraction (SAED) pattern of $CoCr_2O_4$ before and after OER. SAED pattern of $CoCr_2O_4$ in (a) pristine, (b) after 100 CV, and (c) after 1000 CV cycles, additional dots appeared after OER indicating the potential existence of additional phases as marked by red circle with corresponding lattice constants. Specifically, after 100 cycles, the additional dots could be attributed to α-$Co(OH)_2$ (JCPDS No-46-0605), β-CoOOH(JCPDS No-07-0169) or γ-CoOOH (JCPDS No-06-0075); after 1000 cycles, the red circle could be attributed to α-$Co(OH)_2$, β-$Co(OH)_2$ (JCPDS No-30-0443) or β-CoOOH. The fitting details are listed in Supplementary Table S3.

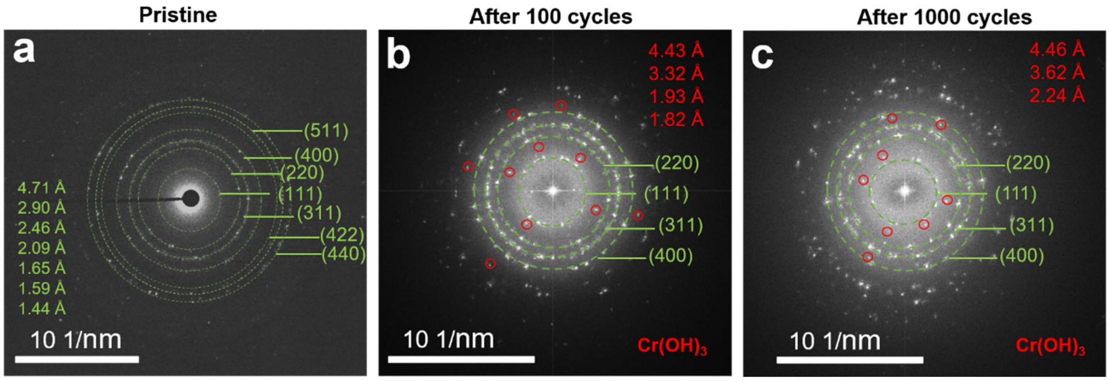

Figure S12. SAED pattern of Co_2CrO_4 in (a) pristine, (b) after 100 CV, and (c) after 1000 CV cycles, additional dots appeared after OER indicating the potential existence of additional phases as marked by red circle with corresponding lattice constants. Specifically, after 100 cycles and 1000 cycles, the additional dots could be attributed to $Cr(OH)_3$. The fitting details are listed in Supplementary Table S4.

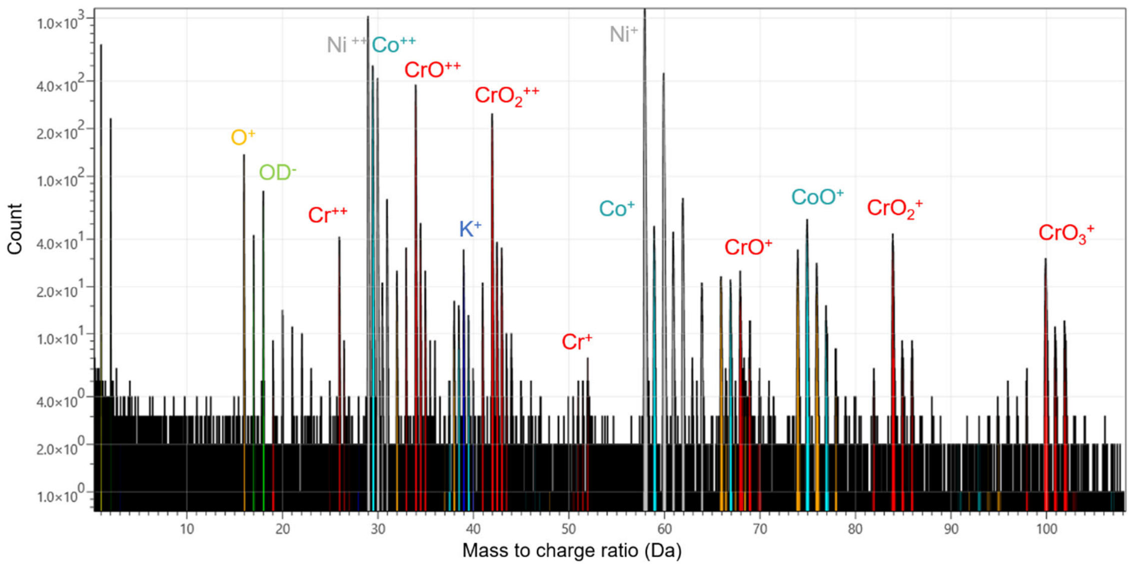

Figure S13. Full mass spectrum collected from a representative $CoCr_2O_4$ nanoparticle after 100 CV cycles.

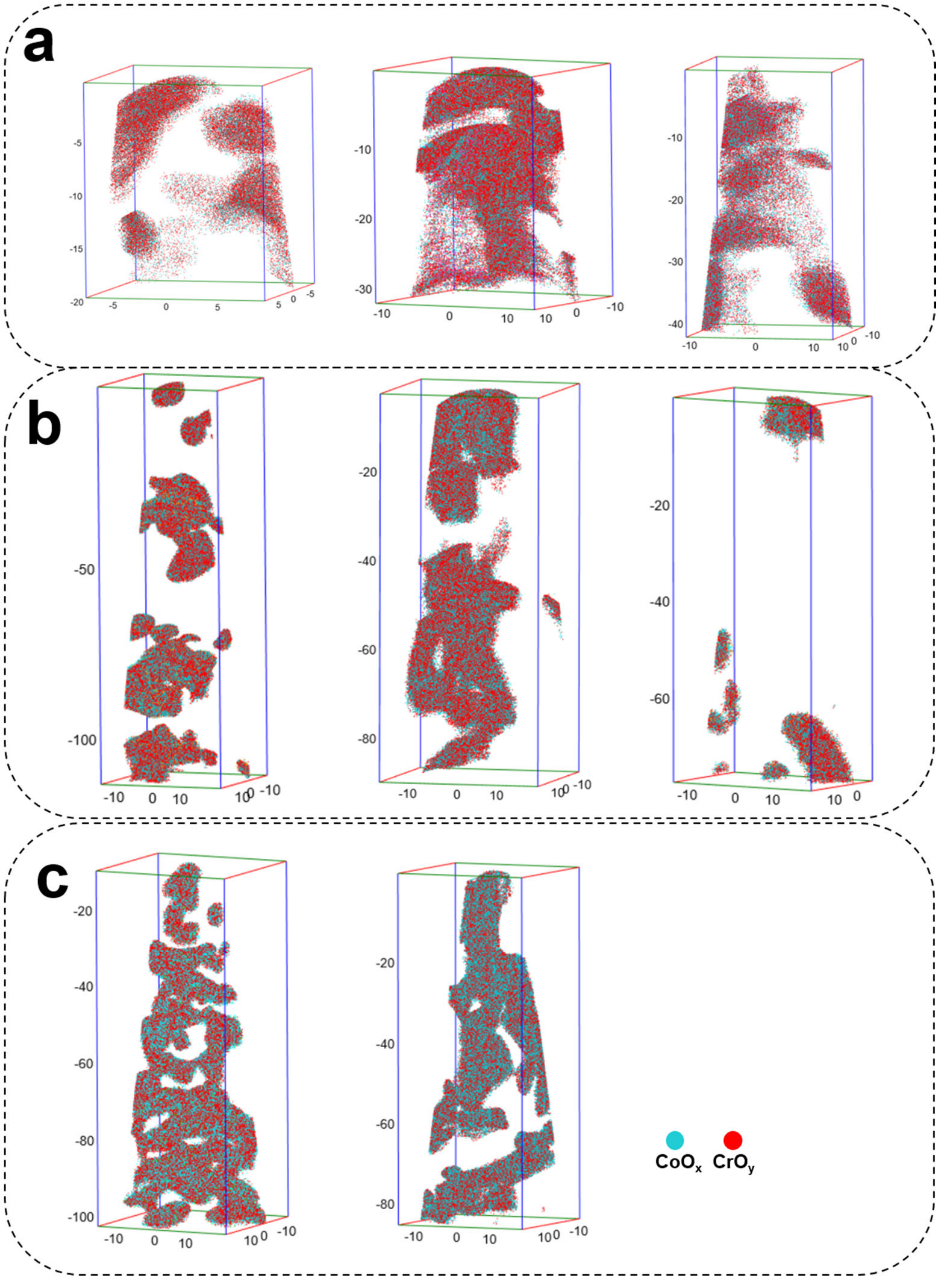

Figure S14. APT data of $CoCr_2O_4$ in pristine and after OER. 3D APT reconstructions of $CoCr_2O_4$ nanoparticles in (a) pristine, (b) after 100 cycles, (c) after 1000 cycles of CV measurements conducted under OER conditions (the Ni matrix is not displayed), acquired by exporting the data within the (Co+Cr) iso-concentration surface.

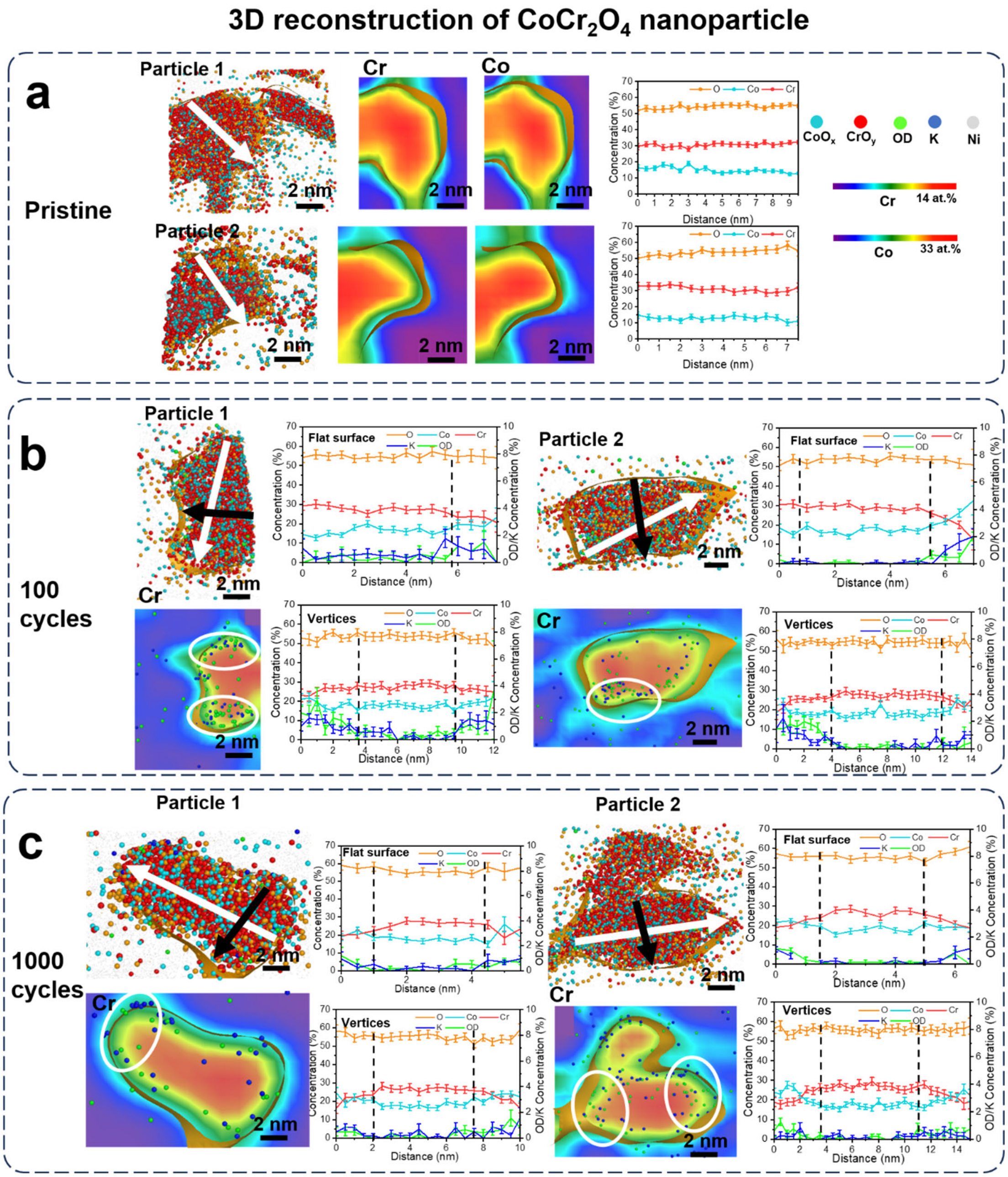

Figure S15. Additional examples of APT reconstruction of $CoCr_2O_4$ in the (a) pristine, (b) after 100 CV cycles, and (c) after 1000 CV cycles. The nanoparticles are embedded in the Ni matrix along with a 2D concentration map of Co and/or Cr as indicated in the figure. The OD-rich regions are indicated in the white circle and 1D concentration profiles are plotted along the white (cover OD and K-rich corners) or black arrows (OD or K-less flat edges) that are marked in the atom distribution map with corresponding arrows marked in the figure. The error bars for the concentration were calculated from $\sqrt{\frac{(100-c)c}{N}}$, where c is the concentration (in at.%) and N is the total number of atoms within the bin of the profile.

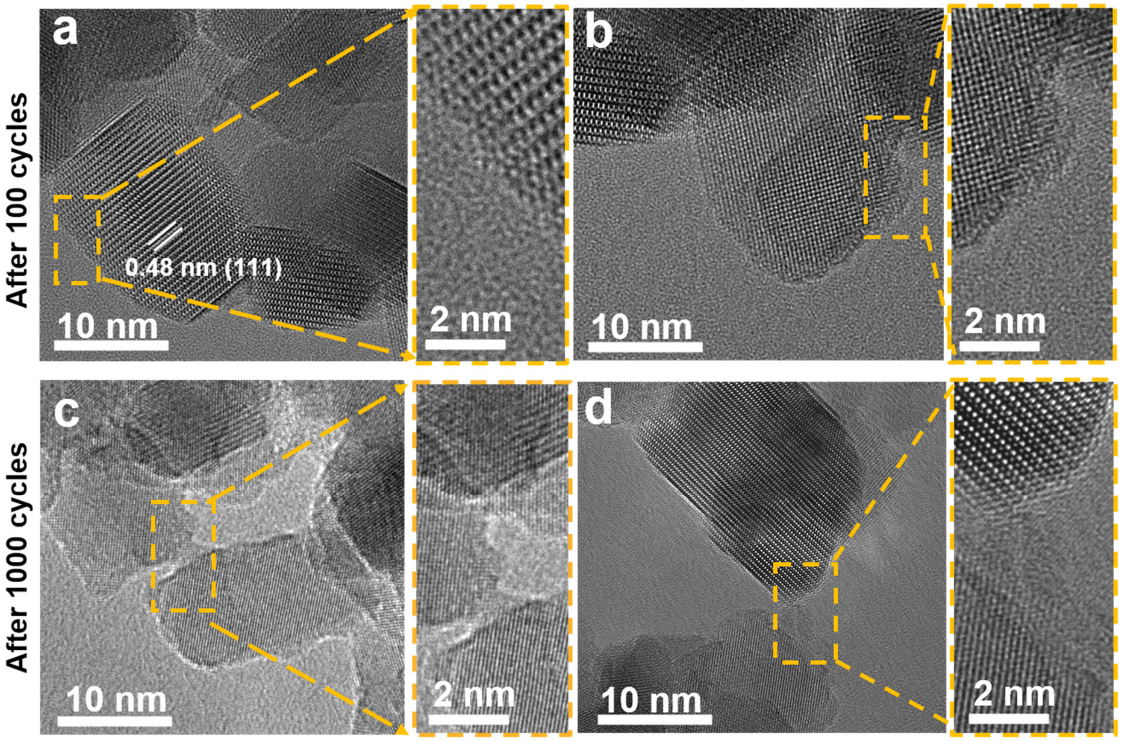

Figure S16. Additional HRTEM pictures of $CoCr_2O_4$ after OER with corresponding zoom-in cubic regions showing the surface evolution. HRTEM images taken from surface regions of $CoCr_2O_4$ after (a, b) 100 cycles and (c, d) 1000 cycles. Small nanoparticles are observed and marked in red cubic which are not observed in pristine and after 100 cycles, indicating the depletion of the nanoparticle during the long-term CV cycles.

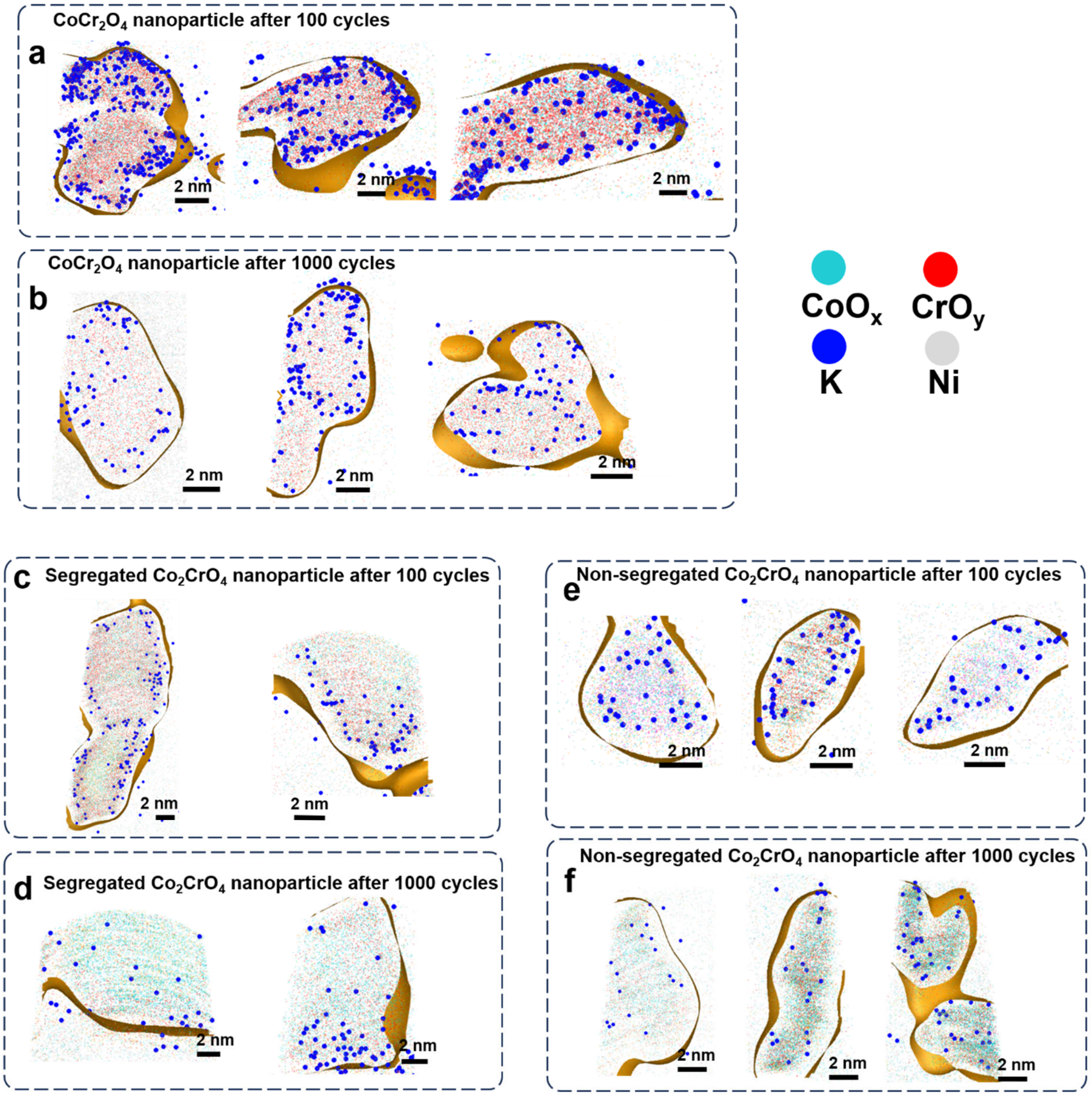

Figure S17. K ion distribution in APT data of $CoCr_2O_4$ and Co_2CrO_4 after OER. Examples show individual nanoparticle reconstructions with K distribution (in blue circle) of $CoCr_2O_4$ in (a) after 100 cycles and (b) after 1000 CV cycles, segregated $CoCr_2O_4$ in (c) after 100 cycles and (d) after 1000 cycles, and non-segregated $CoCr_2O_4$ in (e) after 100 cycles and (f) after 1000 cycles under OER conditions.

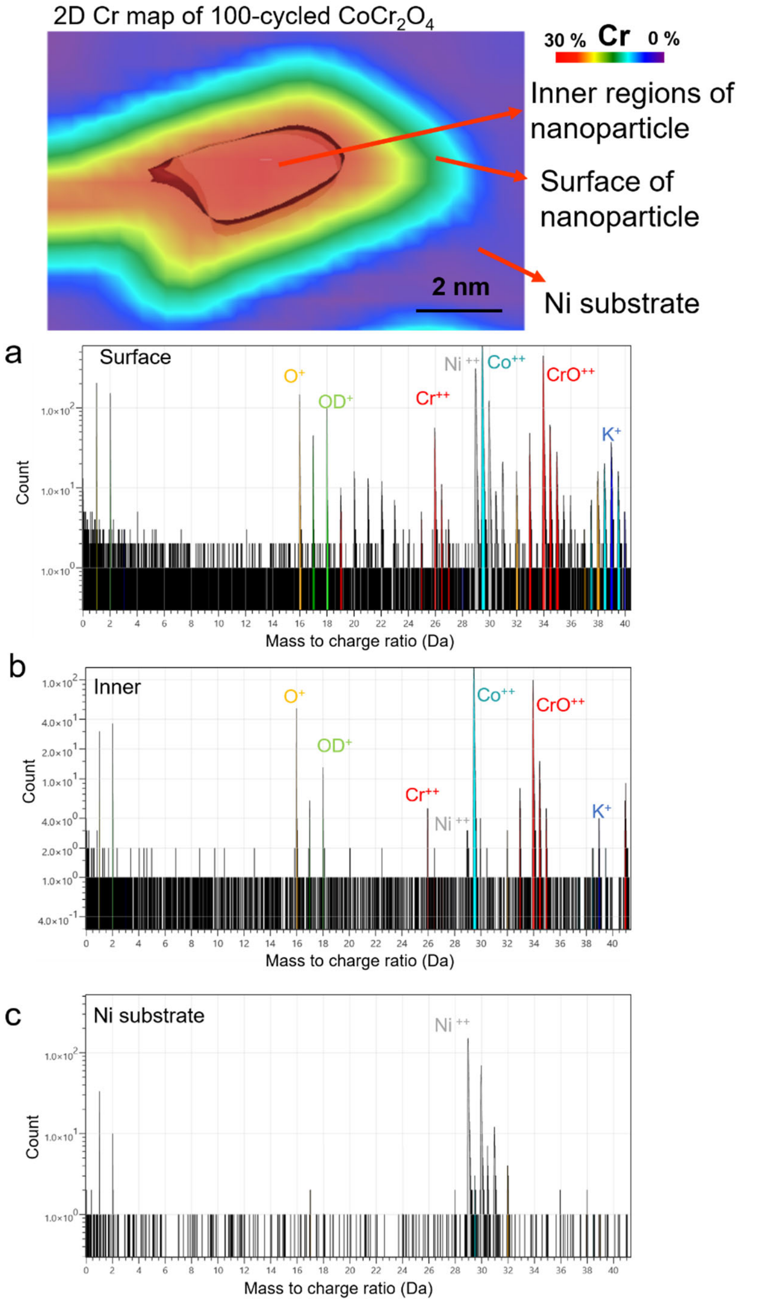

Figure S18. Localized mass spectra extracted from (a) surface of the nanoparticle, (b) inner of the nanoparticle, and (c) Ni substrate in no nanoparticle regions of $CoCr_2O_4$ after 100 cycles within the range of 0-40 Da, all the regions marked in the 2D Cr map of 100-cycle $CoCr_2O_4$ nanoparticles on the top. Figure S18b clearly shows that the interior region of the nanoparticles contains some amounts of K (at 39 Da), hydroxide ions (OD at 18 Da), and water molecules (D_2O at 20 Da), albeit in much smaller quantities than those in the surface/near-surface regions (Figure S18a). Also, the Ni matrix contains nearly no K and OD (Figure S18c), which excludes the possibility that the presence of K and hydroxide ions (OD) originates from their trajectory aberration (APT artefact[1]) from the matrix materials (Ni).

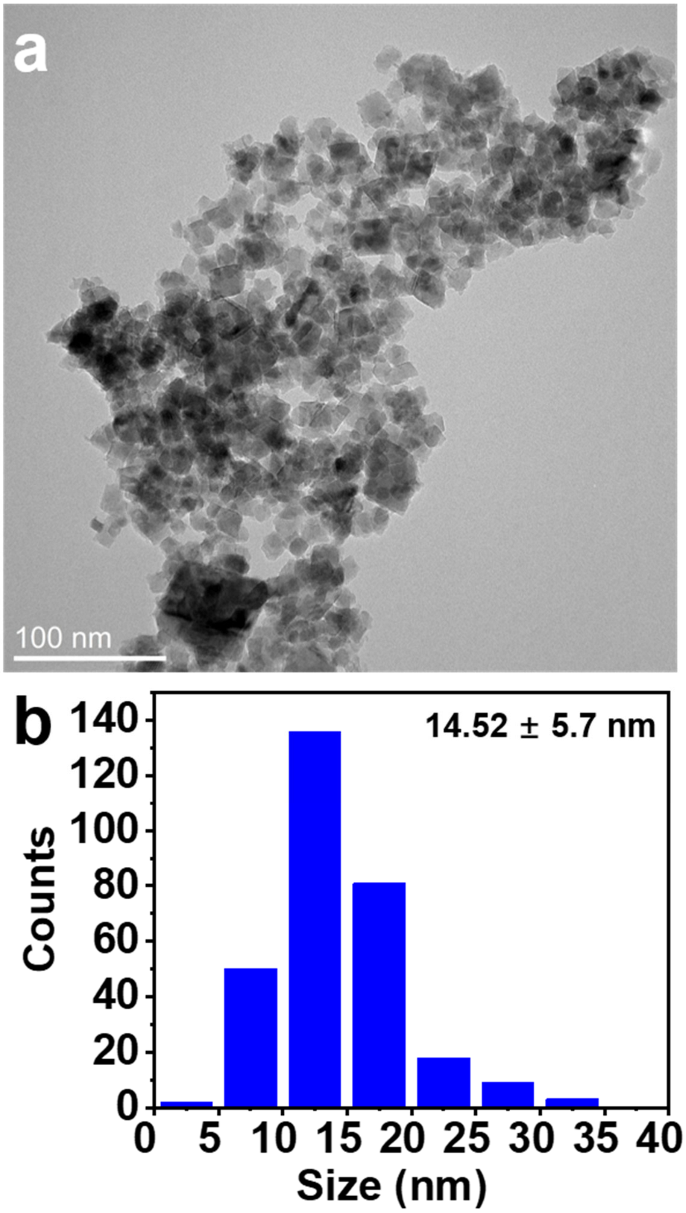

Figure S19. (a) Low-resolution TEM pictures of $CoCr_2O_4$ after 1000 CV cycles with (b) particle size distribution histogram, and the errors are standard deviation. $CoCr_2O_4$ shows a significant decrease in average size compared to that of pristine nanoparticles.

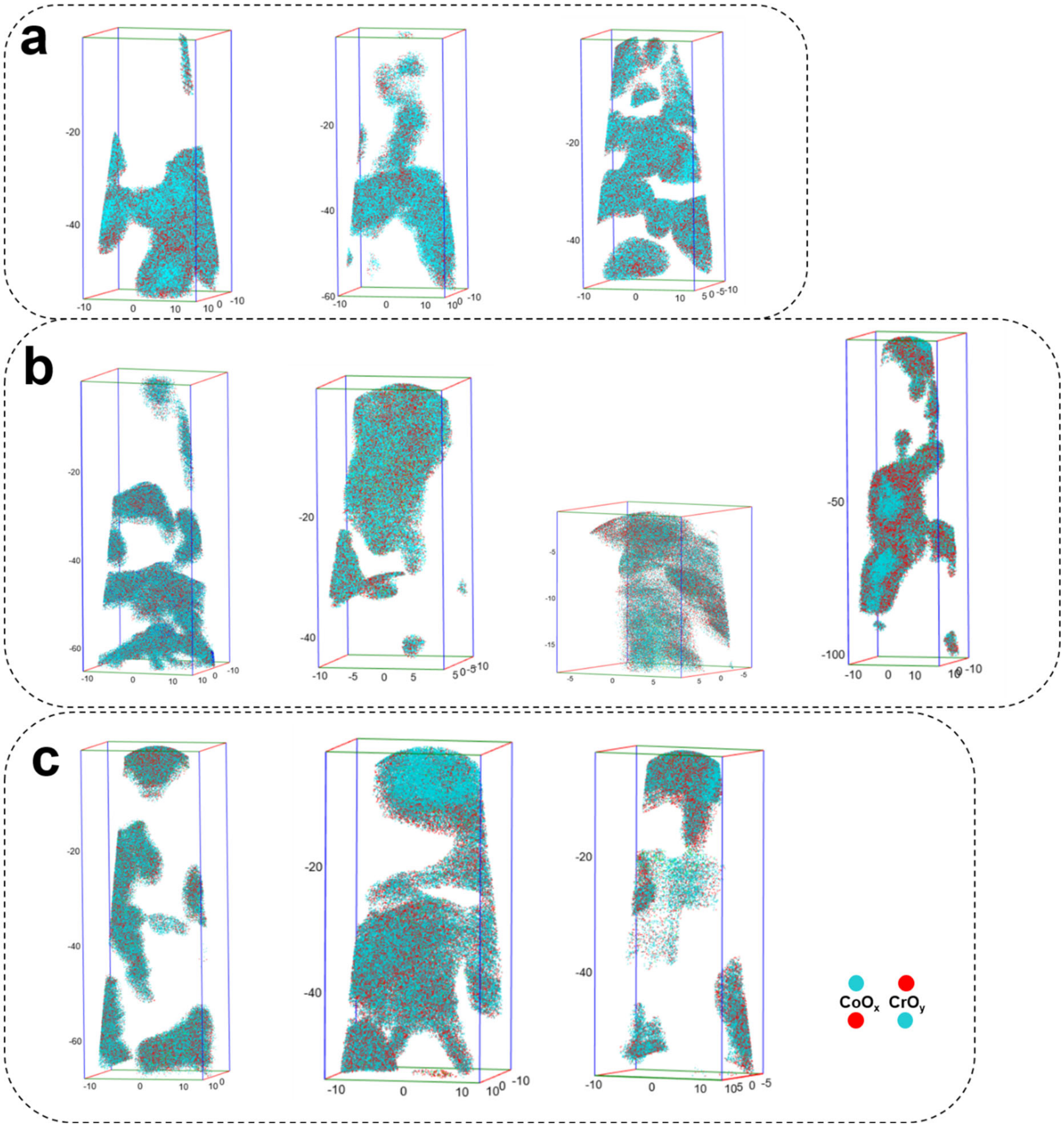

Figure S20. APT data of Co_2CrO_4 in pristine and after OER. 3D APT reconstructions of Co_2CrO_4 nanoparticles in (a) pristine, (b) after 100 cycles, (c) after 1000 cycles of CV measurements conducted under OER conditions (the Ni matrix is not displayed), acquired by exporting the data within the (Co+Cr) iso-concentration surface.

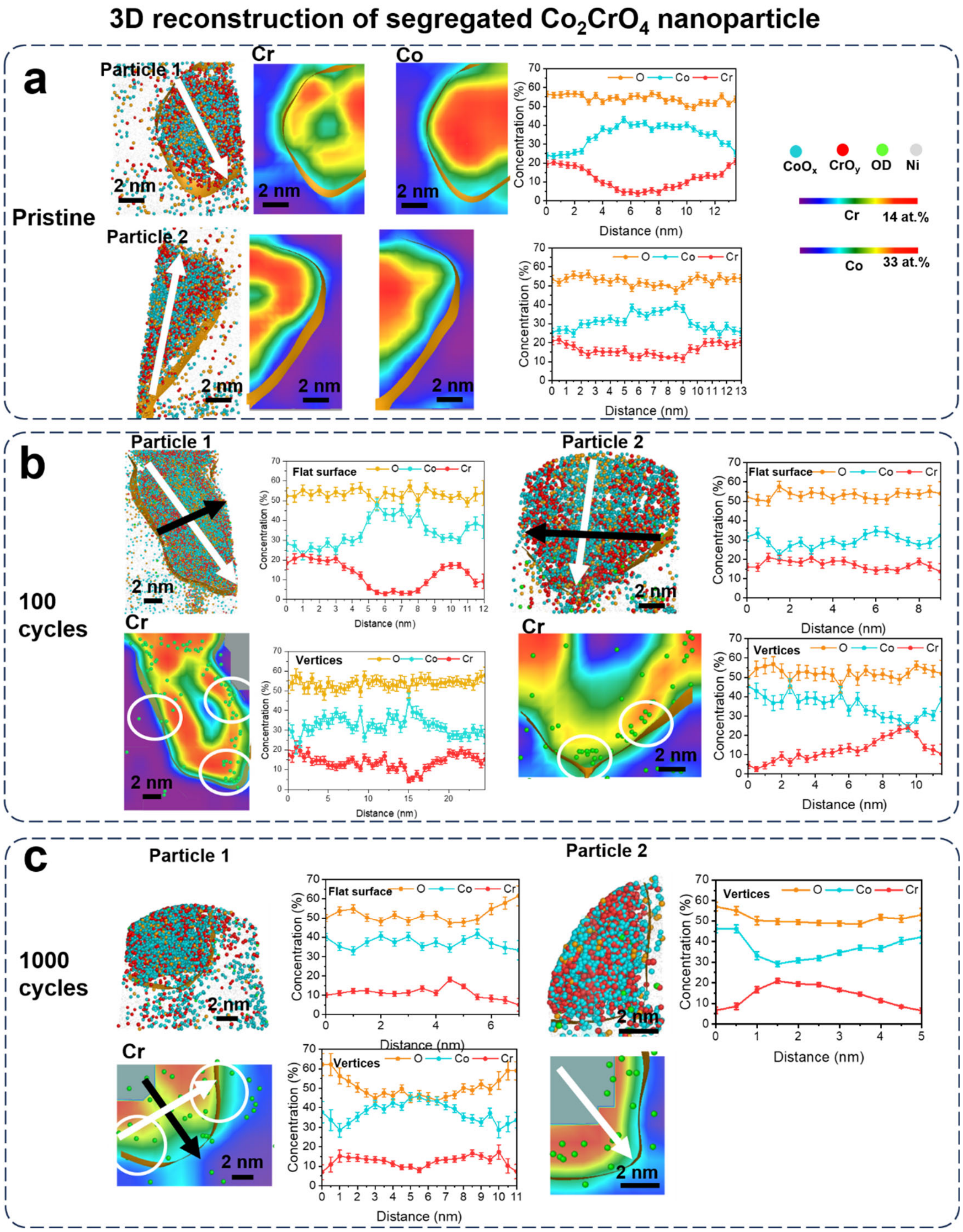

Figure S21. Additional examples of APT reconstruction of segregated-Co_2CrO_4 in the (a) pristine, (b) after 100 CV cycles, and (c) after 1000 CV cycles. The nanoparticles are embedded in the Ni matrix along with 2D concentration map of Cr. The OD-rich regions are indicated in the white circle and 1D concentration profiles are plotted along the white (cover OD-rich corners) or black arrows (OD-less edges) that are marked in the atom distribution map with corresponding arrows marked in the figure. The error bars for the concentration were calculated from $\sqrt{\frac{(100-c)c}{N}}$, where c is the concentration (in at.%) and N is the total number of atoms within the bin of the profile.

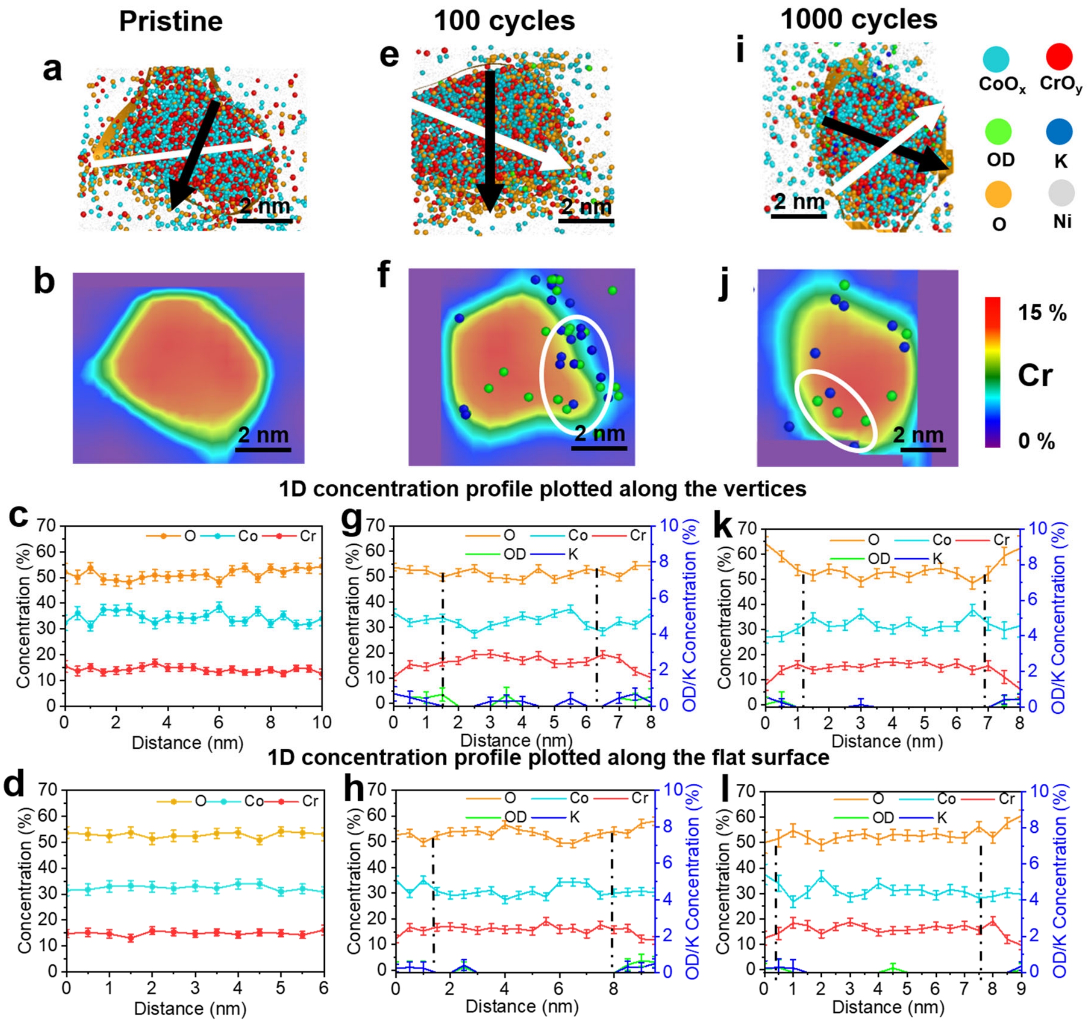

Figure S22. 3D-APT reconstruction of non-segregated-Co_2CrO_4 nanoparticle showing the compositional evolution during OER. a), e), and i) 3D atom maps of non-segregated-Co_2CrO_4 nanoparticles in Ni matrix in the pristine state, after 100, and after 1000 CV cycles, respectively; 2D compositional maps of Cr in b), f) and j), the OD and K distribution at different states after OER are also shown with their rich region marked in the white circle; c), g), k), d), h), and l) show the 1D concentration profiles of non-segregated-Co_2CrO_4, obtained by placing analysis cylinders with a diameter of 4 nm along the arrows with corresponding color marked in the 3D atom maps a), e), and i), in which white arrows from vertex to vertex and black from flat surface edge to edge, revealing the composition of metal composition changes. The error bars for the concentration were calculated from $\sqrt{\frac{(100-c)c}{N}}$, where c is the concentration (in at.%) and N is the total number of atoms within the bin of the profile.

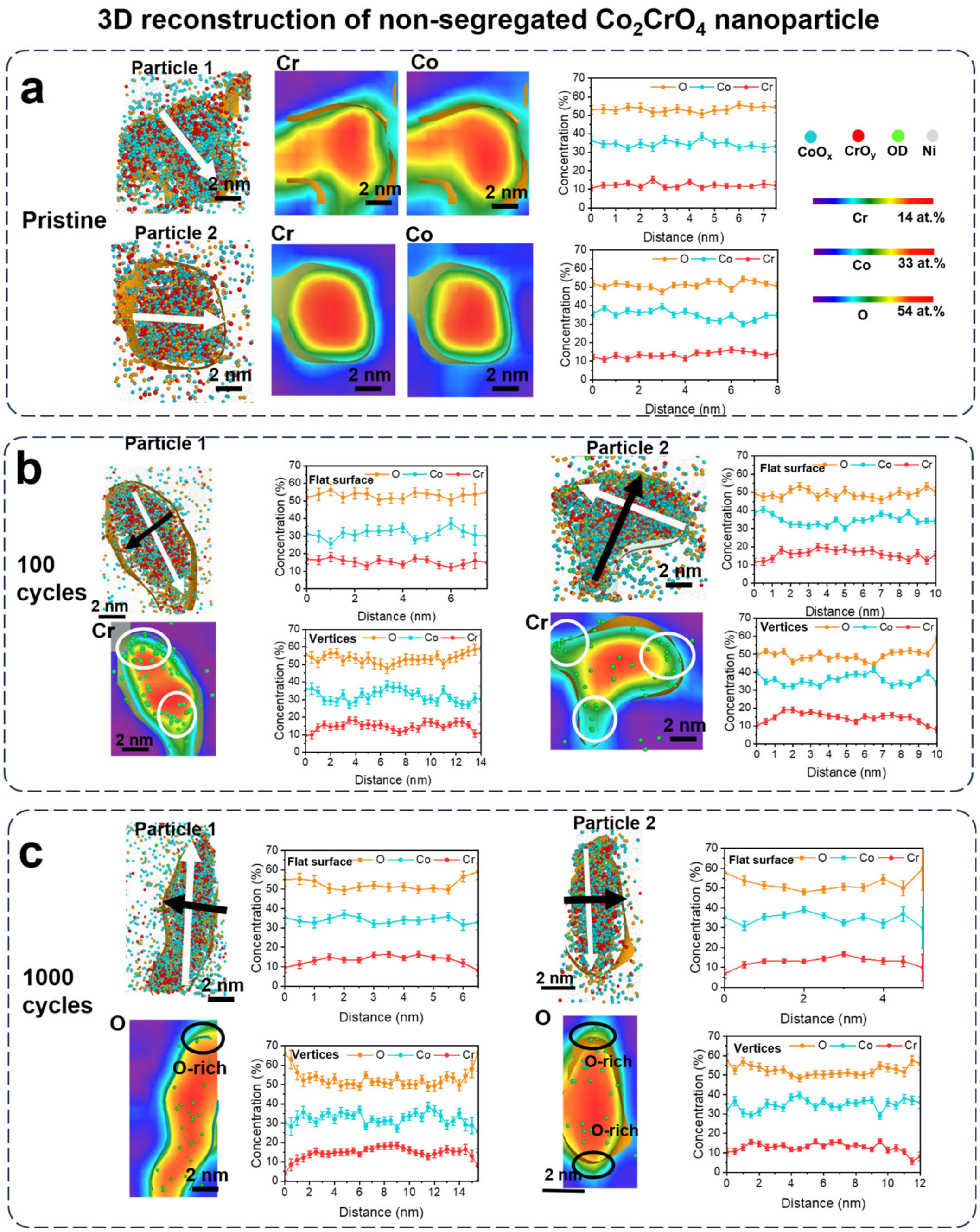

Figure S23. Additional examples of APT reconstruction of non-segregated-Co$_2$CrO$_4$ in the (a) pristine, (b) after 100 CV cycles, and (c) after 1000 CV cycles. The nanoparticles are embedded in the Ni matrix along with a 2D concentration map of Co, Cr, or O as indicated in the figure. The OD-rich regions are indicated in the white circle after 100 cycles, O-rich regions are indicated in the black circle after 1000 cycles, and 1D concentration profiles are plotted along the white (cover OD-rich corners) or black arrows (OD-less edges) that are marked in the atom distribution map with corresponding arrows marked in the figure. The error bars for the concentration were calculated from $\sqrt{\frac{(100-c)c}{N}}$, where c is the concentration (in at.%) and N is the total number of atoms within the bin of the profile.

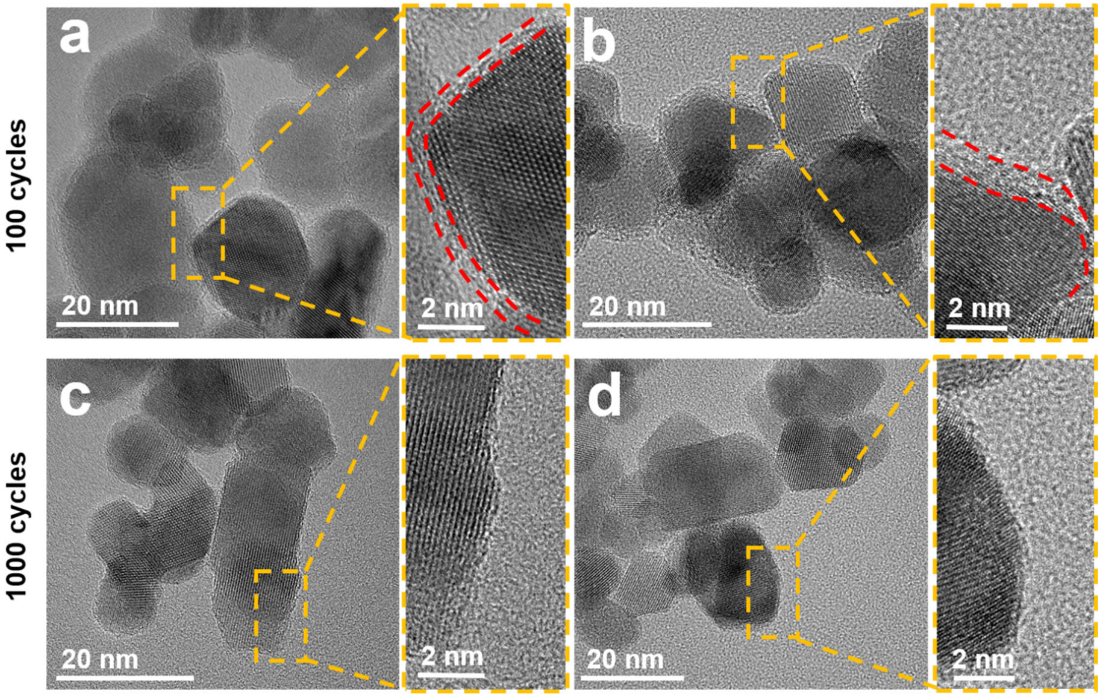

Figure S24. Additional HRTEM pictures of Co_2CrO_4. (a, b) after 100 cycles, and (c, d) after 1000 cycles with corresponding zoom-in cubic regions showing the surface evolution.

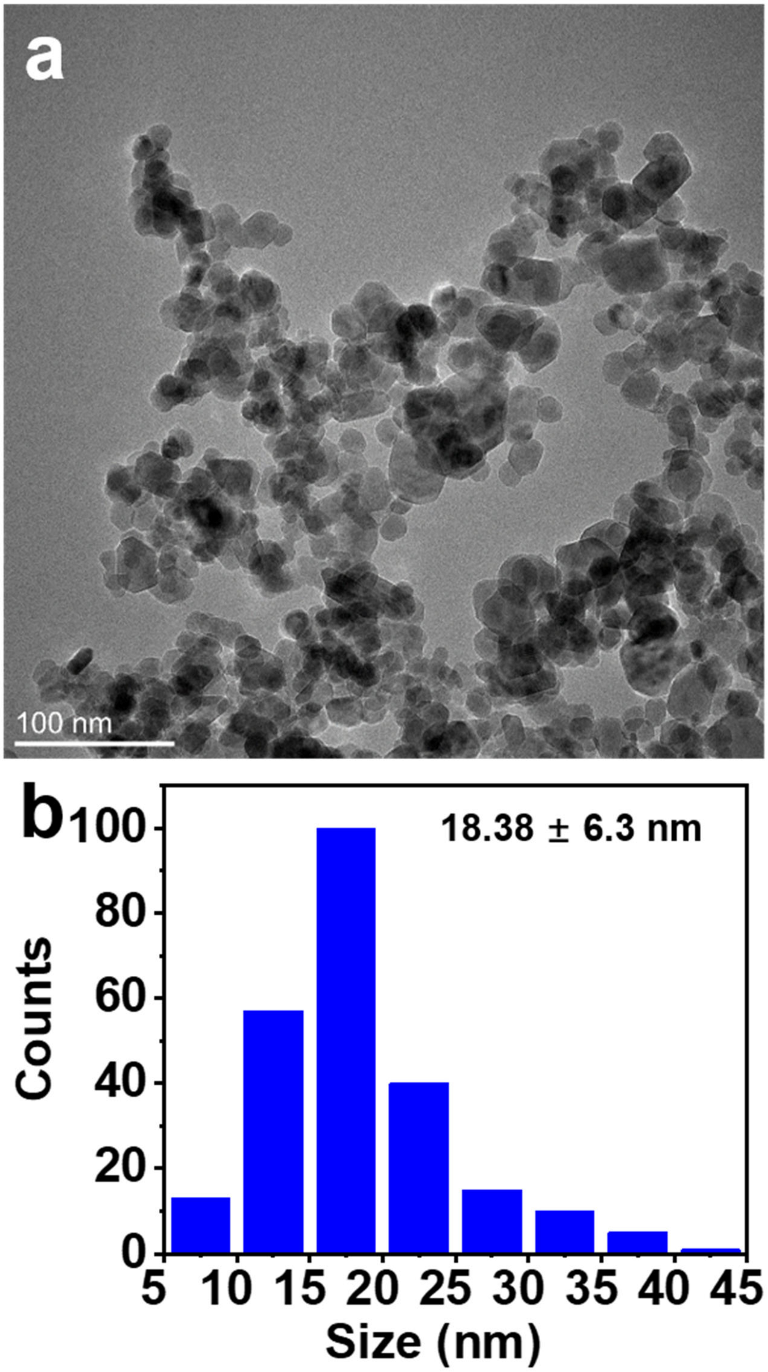

Figure S25. (a) Low-resolution TEM pictures of Co_2CrO_4 after 1000 CV cycles with (b) particle size distribution histogram, and the errors are standard deviation.

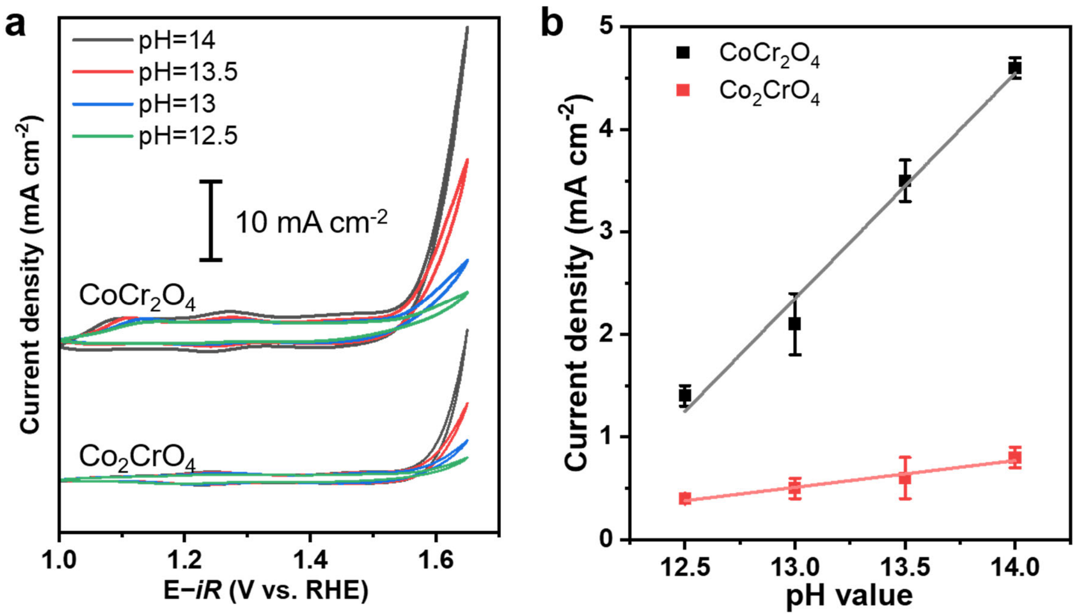

Figure S26. (a) CV measurements from O_2-saturated KOH with different pH values ranging from 12.5-14.0±0.1 recorded at a scan rate of 20 mV s^{-1}; (b) Specific OER activity at 1.55 V vs. RHE at different pH, and the error bars represent standard deviation of three repeated experiments. All CV measurements are conducted in KOH electrolyte with different concentration, and on 0.196 cm^2 glassy carbon electrode with a mass loading of ~0.05 mg at room temperature. The rotation speed is 1600 rpm and the potential range is 1-1.65 V, and the compensation resistances are done automatically by the potentiostat.

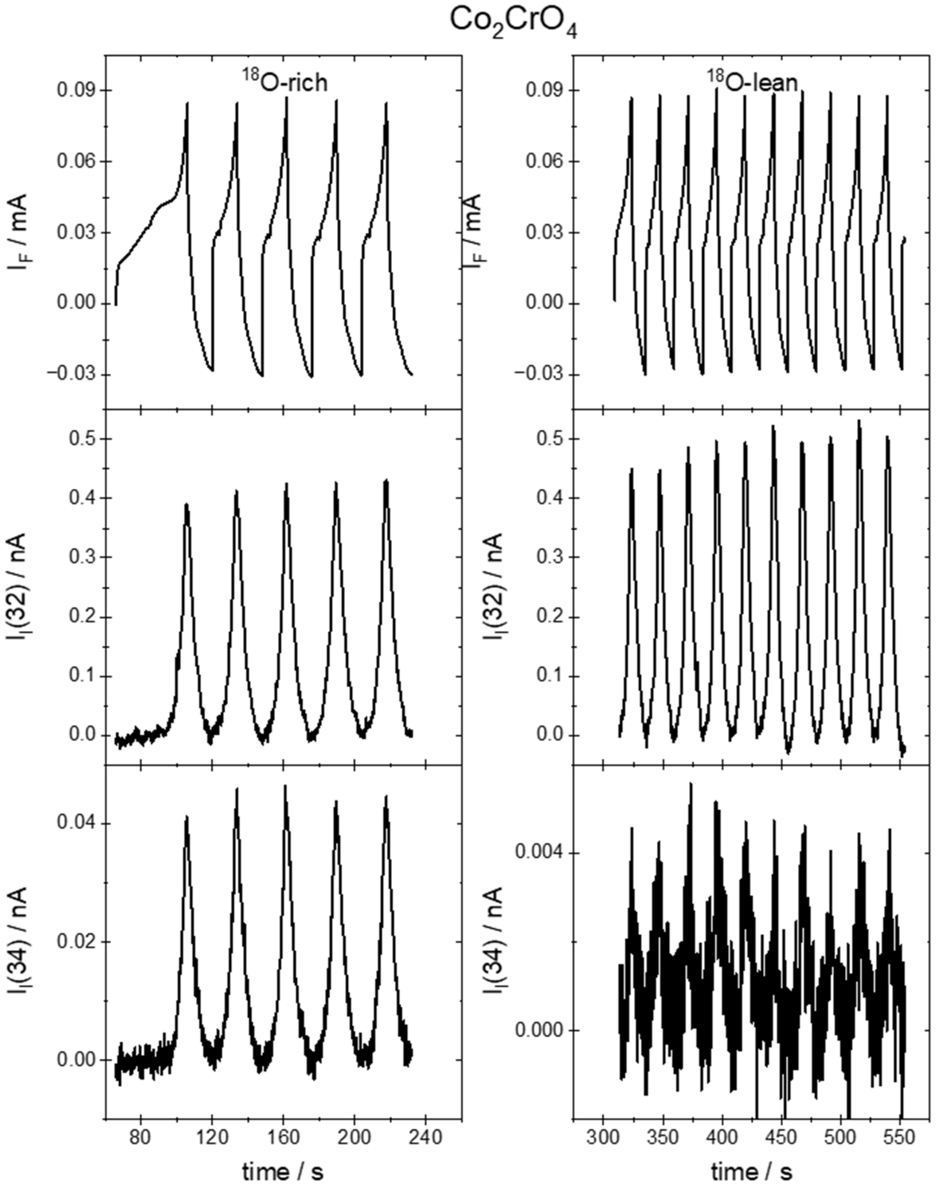

Figure S27. DEMS results of the electrolyte exchange experiment conducted at Co_2CrO_4. Top Panels: Faradaic current measured by the potentiostat. Centre: Mass spectrometric signal for mass 32. Bottom: Mass spectrometric signal for mass 34. The mass spectrometer measures the signal in current, but Faradays laws are not applicable. Left: measured in 1 M KOH featuring 5% $H_2{}^{18}O$. Right: measured in 1 M KOH in normal water featuring with natural isotope abundance. Conditions: 5 mV/s, electrolyte flow rate 5 μL/s. The electrolyte exchange was performed at 0.53 V vs. Hg/HgO and took 78 seconds. Prior to the experiment Co_2CrO_4 was activated by performing 100 cycles in 1 M KOH electrolyte with normal water.

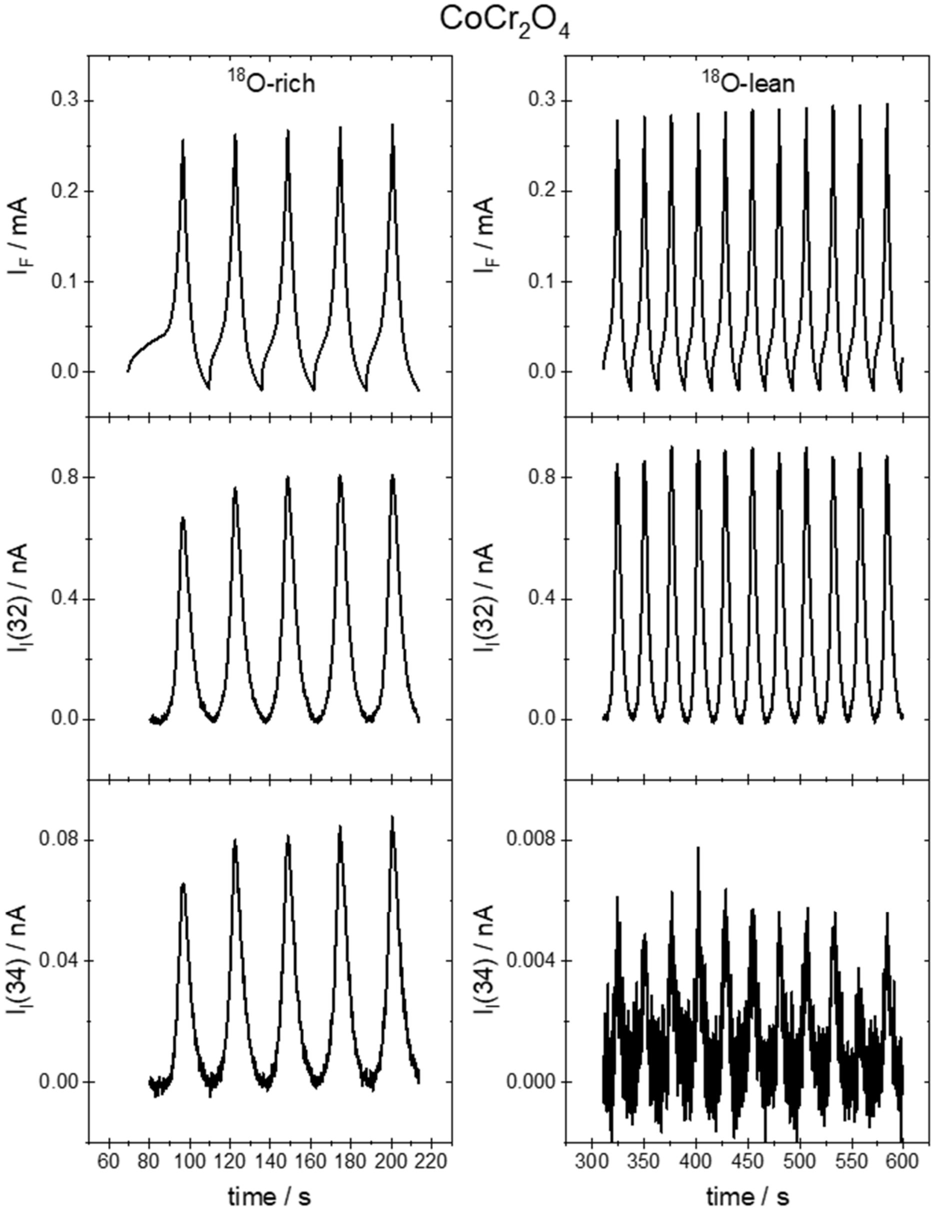

Figure S28. DEMS results of the electrolyte exchange experiment conducted at $CoCr_2O_4$. Top Panels: Faradaic current measured by the potentiostat. Centre: Mass spectrometric signal for mass 32. Bottom: Mass spectrometric signal for mass 34. The mass spectrometer measures the signal in current, but Faradays laws are not applicable. Left: measured in 1 M KOH featuring 5% $H_2{}^{18}O$. Right: measured in 1 M KOH in normal water featuring with natural isotope abundance. Conditions: 10 mV/s, electrolyte flow rate 5 µL/s. The electrolyte exchange was conducted at 0.54 V vs. Hg/HgO and took 98 seconds. Prior to the experiment $CoCr_2O_4$ was activated by performing 100 cycles in the 1 M KOH electrolyte with normal water.

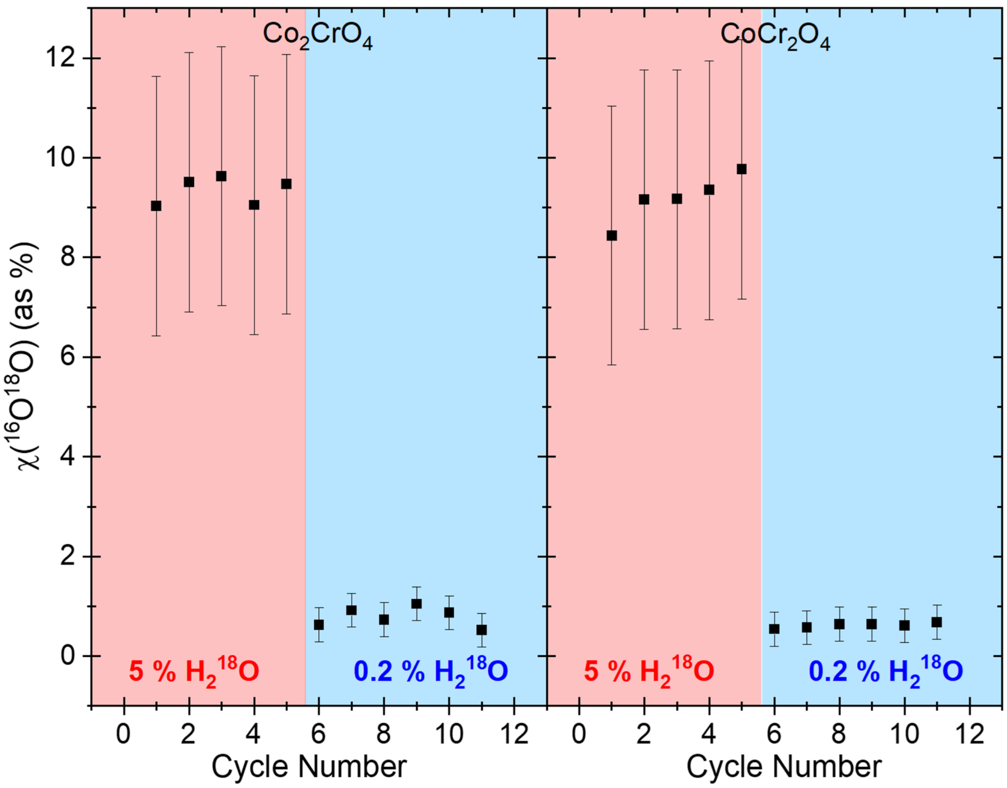

Figure S29. $\chi(^{16}O^{18}O)$ changes with cycles, which defined as the fraction of $^{18}O^{16}O \cdot 100 / (^{18}O^{16}O + ^{16}O^{16}O)$ versus the cycle number. Between the 5th and the 6th the exchange from the electrolyte featuring 5% $H_2{}^{18}O$ to the ^{18}O-lean electrolyte with natural abundance is performed. $\chi(^{16}O^{18}O)$ was determined from the experimental values shown in Figure S27 and Figure S28, respectively. The errors are standard deviation from three repeated measurement.

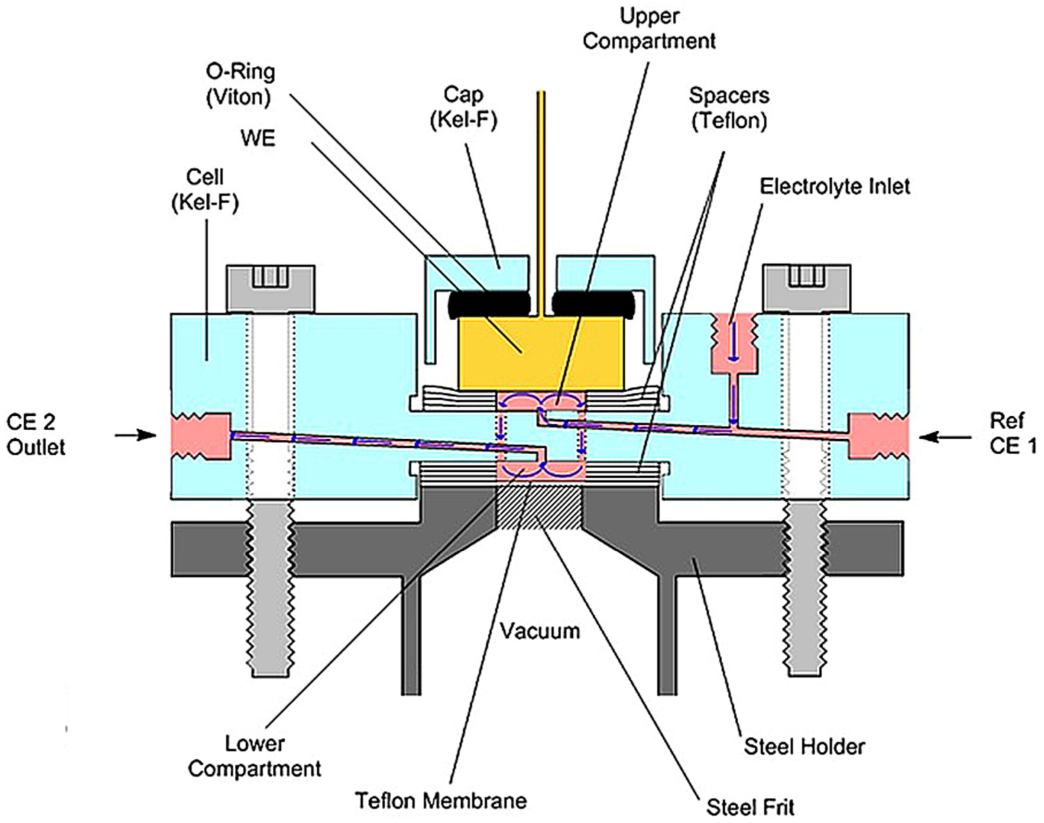

Figure S30. Schematic figure of the Dual Thin Layer Cell used for DEMS, copied with Permission under Creative Commons CC-BY License from Bondue et al.,[2] Copyright 2020 Elsevier B.V. Blue arrows indicate the flow of the electrolyte. The electrolyte is stored in a reservoir connected to the inlet. From there, it enters the center of the upper chamber, a hollow space created by pressing the working electrode (WE) against a Teflon spacer. It then passes through six radially arranged capillaries into the lower chamber, which is shaped by the steel holder compressed against Teflon spacers. A Teflon membrane separates the electrolyte from the steel holder and simultaneously establishes the vacuum-electrolyte interface. A 100 Ω resistor connects the potentiostat to the second counter electrode (CE 2) at the outlet, which carries the majority of the current. To channel a fraction of the current toward the reference electrode (Ref), the potentiostat is also linked to the first counter electrode (CE 1) via a 100 kΩ resistor.

Supplementary Table 1. The concentration of O, Cr, and Co in both nanoparticles obtained from TEM/EDS.

EDS	O %	Cr %	Co %
$CoCr_2O_4$	63.5 ± 0.4	22.5 ± 0.6	14.1 ± 0.3
Co_2CrO_4	58.2 ± 0.7	12.7 ± 0.7	29.1 ± 0.6

Supplementary Table 2. EIS simulation results obtained by using the equivalent circuit model with Zviwer software.

Samples	$CoCr_2O_4$	$CoCr_2O_4$ after 100 cycles	$CoCr_2O_4$ after 1000 cycles	Co_2CrO_4	Co_2CrO_4 after 100 cycles	Co_2CrO_4 after 1000 cycles
Rs (Ω)	10.36	10.11	10.29	10.9	10.87	10.85
Rct (Ω)	10.49	7.01	12.1	27.92	34.25	58.07
CPE1 (10^{-3}F)	5.7	7.5	7.5	1.9	0.8	0.5
Error	0.09	0.02	0.4	0.2	0.6	0.6

Supplementary Table 3. Fitting results of the additional dots in the SAED pattern of $CoCr_2O_4$ after OER.

Phase structure	After 100 CV cycles			After 1000 CV cycles		
	2.36 Å	1.92 Å	1.71 Å	1.93 Å	1.73 Å	1.54 Å
α-$Co(OH)_2$	(015)	(108)	(110)		(102)	(111)
β-$Co(OH)_2$	(101)		(102)	(108)		(110)
β-CoOOH	$(01\bar{1}2)$	$(10\bar{1}4)$	$(0\bar{1}15)$	$(10\bar{1}4)$	$(10\bar{1}5)$	$(10\bar{1}7)$
γ-CoOOH	$(10\bar{1}2)$	$(10\bar{1}5)$	$(01\bar{1}8)$			$(10\bar{1}7)$

Supplementary Table 4. Fitting results of the additional dots in the SAED pattern of Co_2CrO_4 after OER.

Phase structure	After 100 CV cycles				After 1000 CV cycles		
	4.43 Å	3.32 Å	1.93 Å	1.82 Å	4.46 Å	3.62 Å	2.24 Å
α-$Co(OH)_2$	(006)		(018)		(006)		
β-$Co(OH)_2$	(001)		(102)		(001)		(002)
β-CoOOH	(0003)		$(10\bar{1}4)$	$(01\bar{1}5)$	(0003)		(0006)
γ-CoOOH		(0006)				(0006)	
CrOOH	(0003)		$(10\bar{1}4)$	$(01\bar{1}5)$	(0003)		(006)
$Cr(OH)_3$	(100)	(101)	(201)	(112)	(100)	(101)	(102)

Supplementary Note 1: S number calculation

In addition, the stability number (S number) can be obtained from the ICP-MS results which are used as a metric to evaluate the activity and stability relationship.[3] The S number is defined as the ratio between the number of moles of oxygen generated and the number of moles of active metal dissolved during water electrolysis. By estimating the number of moles of evolved oxygen by integrating the current during the 100 CV cycles, the amount of oxygen can be determined by $Q = \frac{1}{zF}\int i(t)\ \mathrm{d}t$, in which z is the number of moles of electrons transferred during the OER (z = 4), F is the Faraday constant, and t is the duration of the CV (1 - 1.65V vs. RHE) test. Note that the metal redox and capacitance in non-OER regions during CV also contributed to the current, the calculation is used for approximate estimation of the stability. Here, Co is considered as the active site for both nanoparticles, and the dissolved amounts of Co are 0.2924 μg and 0.577 μg for $CoCr_2O_4$ and Co_2CrO_4, respectively. The total numbers of transferred electrons (Q) after the 1000 cycles of CV are 8.35 C for $CoCr_2O_4$ and 2.35 C for Co_2CrO_4, respectively. The S-number is calculated as $S = \frac{QM_{Co}}{4Fm_{Co}}$, where M_{Co} is the mole mass of Co and m is the cumulative amount loss of Co obtained from the ICP-MS data. Then 4.3×10^4 for $CoCr_2O_4$ and 6.2×10^3 for Co_2CrO_4 is obtained, showing better OER stability of $CoCr_2O_4$.

Supplementary Note 2: XPS Section

The spectra were analyzed using the software CasaXPS version 2.3025R1.0. A standard Shirley background was applied to all spectra. The spectra were charge calibrated by setting the C 1*s* peak to 284.8 eV or 284.5 eV depending on whether the carbon signal originated from adventitious carbon or the exposed glassy carbon substrate. To fit the Co $2p_{3/2}$, Cr $2p_{3/2}$, and O 1*s*, a Gaussian-Lorentzian product formula GL(m) was used to describe the line shape of the peaks in CasaXPS with m describing the mixing and, for example, m = 30 being a 30% Lorentzian and 70% Gaussian line shape. All spectra were recorded with a pass energy of 200 eV in fixed transmission mode. To analyze the Co 2*p* and Cr 2*p* regions, an approach using peak models of reference models based on the work of Biesinger et al. was used to account for the multiplet splitting and assign chemical states. The peak models were constructed based on literature spectra of the Co $2p_{3/2}$ and Cr $2p_{3/2}$ of different cobalt and chromium species and the peaks were constrained regarding their relative peak positions, FWHM, and area ratios to the first peak in the respective model. This peak was additionally constrained in position (±0.2 eV from literature model) and FWHM (literature value to literature value +0.5 eV. Models of Co(0), CoO, $Co(OH)_2$, CoOOH, Co_3O_4 were considered for the fitting of the Co $2p_{3/2}$[4, 5] For the analysis of the Cr $2p_{3/2}$, peak models of Cr(0), Cr_2O_3, $Cr(OH)_3$ and CrO_3 were considered.[5] In addition, the Co 2*p* and Cr 2*p* spectra of the pristine $CoCr_2O_4$ nanoparticles were used to construct peak models based on a purely empirical fitting assuming that the surface of the nanoparticles consists only of the expected metal oxide species. The peak models were constrained similarly to the models based on literature, but the FWHM of the first peak was constrained to ±0.2 eV of the reference spectra. These were then employed together with the literature models to analyze the spectra of the $CoCr_2O_4$ sample after 1000 cycles. This approach could not be realized for the Co_2CrO_4 nanoparticles as other techniques revealed that their surface contained CoO and was Cr enriched. Instead, the state of cobalt was only approximated from the Co 2*p* using the models mentioned above. The best fit was achieved with a mixture of CoO and Co_3O_4. The share of CoO model in the fits exceeds the difference in Co^{II}/Co^{III} between Co_3O_4 (1:2) and Co_2CrO_4 (1:1) suggesting the presence of segregated CoO. Due to the surface enrichment of Cr on the Co_2CrO_4 nanoparticle surface, no reference model for the fitting of the Cr 2*p* after 1000 cycles could be constructed. In pristine Co_2CrO_4, the Cr $2p_{3/2}$ spectrum could not be fitted using any reference due to the lack of a well-defined structure, with the broad profile suggesting a mixed chemical environment. After 1000 cycles, the spectrum becomes narrower and can be fitted using the empirical model from $CoCr_2O_4$, indicating that Cr adopts a similar coordination environment. Monte Carlo simulations as implemented in CasaXPS were used to calculate standard deviations (σ) of the quantification and ascertain the accuracy of the fit. After the extended electrochemical treatment of the samples, the sample with $CoCr_2O_4$ had a heterogeneous nanoparticle coverage presumably due to detachment during electrocatalysis. The survey spectra show no contamination aside from a K 2*p* signal that was observed on the electrochemically treated $CoCr_2O_4$ sample possibly due to residual KOH.

Supplementary Note 3: OD counts calculation details

For the mass spectrometry analysis in which the mass-to-charge (m/z) ratios between 17 and 19 Da are being investigated for OD counts calculation after different cycles (100 and 1000). The peaks at 17, 18, and 19 Da are interpreted as products from oxygen and hydrogen interactions within the vacuum chamber, resulting in molecular ions like OH^+, OH_2^+, and OH_3^+.

To resolve and quantify the presence of OD^+ (which may overlap with the OH_x^+ peaks at 18 and 19 Da), the analysis includes:

- The peak at **18 Da** is composed of OH_2^+ and OD^+.
- The peak at **19 Da** contains OH_3^+ and ODH^+.

To separate OD counts from OH counts at 18 and 19 Da, the researchers rely on the peak at **17 Da** and known ratios of hydrogen ion species (H^+, H_2^+, and H_3^+) to estimate the contribution of OH_2^+ and OH_3^+. The count for OH_2^+ at 18 Da is calculated using the formula:

$$\mathrm{OH_2^+ \text{ count at 18 Da}} = \frac{\mathrm{N_{m/z=17}} \cdot \mathrm{N_{m/z=2}}}{\mathrm{N_{m/z=1}}}$$

The remaining counts at 18 Da are attributed to OD^+. Similarly, the OH_3^+ counts at 19 Da can be calculated using a similar method.

Supplementary Note 4: DEMS measurements

Isotope-labeled operando DEMS measurements were performed on $CoCr_2O_4$ and Co_2CrO_4 to investigate the reaction mechanisms regarding the lattice oxygen involvement (see Figure S27-S29). The 100-cycle electrocatalytic activation pretreatments were carried out inside DEMS in 1 M KOH electrolyte (in normal H_2O, pH=14.0±0.1). Figures S27 and S28 show the results of the DEMS measurement that were combined with an exchange from a 5% $H_2{}^{18}O$ isotope-labelled electrolyte (^{18}O-rich electrolyte) to the normal water with 0.2% $H_2{}^{18}O$ natural isotope abundance (^{18}O-lean electrolyte, normal water). The upper panels in Figure S27 and S28 show the faradaic current measured by the potentiostat. 15 cycles were recorded in the electrolyte featuring 5% $H_2{}^{18}O$ (^{18}O-rich electrolyte). The left side of Figures S27 and S28 shows only the last 5 cycles before the exchange to the ^{18}O-lean electrolyte, and the right side shows the results in normal water (^{18}O-lean electrolyte). The panels in the middle show the mass spectroscopic response (i.e. ionic current) for mass 32, which is proportional to the formation rate of $^{16}O_2$ and the bottom panels show the mass spectroscopic response for mass 34, which is proportional to the formation rate of $^{16}O^{18}O$. Figure S29 summarizes the molar fraction of $\chi(^{16}O^{18}O)$ changes as a function of the cycle number when the electrolyte was changed from ^{18}O-rich (5% $H_2{}^{18}O$) to ^{18}O-lean (normal H_2O) electrolyte at various cycles. To this end the mass spectroscopic signals for mass 32 and 34 were integrated. $\chi(^{16}O^{18}O)$ was then calculated by dividing the integral for the mass spectroscopic signal for mass 34 by the sum of mass 34 and 32. Mass 36 ($^{18}O^{18}O$) was not measured as it yields ~0.25% molar fraction using 5% $H_2{}^{18}O$ isotope-labelled electrolyte, which is insufficient for reliable evaluation due to high statistical error. The schematic illustration in Figure S30 showing the configuration of the Dual Thin Layer Cell used for DEMS are presented.

In Figure S29, the molar fraction $\chi(^{16}O^{18}O)$ for 100-cycle Co_2CrO_4 is ~0.9% and ~0.6% for 100-cycle $CoCr_2O_4$, slightly higher than ~0.4, which is the theoretical value of $\chi(^{16}O^{18}O)$ from the normal water with 0.2% $H_2{}^{18}O$ natural abundance.[6, 7] This suggests that lattice oxygen mechanisms might occur in both samples. Because ^{18}O-containing species would cover the surfaces of electrocatalysts in 5% $H_2{}^{18}O$ isotope-labelled electrolyte before cycling in normal water. Such ^{18}O-containing species would generate a molar fraction of $^{18}O^{16}O$ (m/z = 34) larger than ~0.4.[6] As OER proceeds in the normal water, the amount of oxygen that contains ^{18}O is expected to decrease due to the gradual consumption of the ^{18}O-containing species on the electrocatalyst surfaces. However, the values of $\chi(^{16}O^{18}O)$ do not decrease steadily in the normal electrolyte and it drops suddenly from 5% $H_2{}^{18}O$ electrolyte to normal water (0.2% $H_2{}^{18}O$), inferring that lattice oxygen might not be involved dominantly. Also, the $\chi(^{16}O^{18}O)$ for 100-cycle $CoCr_2O_4$ is close ~0.4% with the error bars. This makes us refrain from concluding that lattice oxygen mechanisms occur. One possible explanation is that 100-cycle $CoCr_2O_4$ is activated via the (de)intercalation processes. In this case, the oscillations exceeding 0.4% could also likely be attributed to the disproportionate release of residual hydroxide ions and water molecules trapped within the interlayer during the labelling process in ^{18}O-rich electrolyte. Additionally, the transition or dynamics from hydroxide to active oxyhydroxide on 100-cycle $CoCr_2O_4$, facilitated by intercalation, could be interrupted, or some of the active species may be removed during electrolyte exchange. Therefore, we refrain from making conclusive statements based solely on DEMS measurements.

References:

1. Vurpillot F, Bostel A, Blavette D. Trajectory overlaps and local magnification in three-dimensional atom probe. *Applied Physics Letters* **76**, 3127-3129 (2000).

2. Bondue CJ, Koper MT. A DEMS approach for the direct detection of CO formed during electrochemical CO2 reduction. *Journal of Electroanalytical Chemistry* **875**, 113842 (2020).

3. Geiger S*, et al.* The stability number as a metric for electrocatalyst stability benchmarking. *Nature Catalysis* **1**, 508-515 (2018).

4. Yang J, Liu H, Martens WN, Frost RL. Synthesis and characterization of cobalt hydroxide, cobalt oxyhydroxide, and cobalt oxide nanodiscs. *The Journal of Physical Chemistry C* **114**, 111-119 (2010).

5. Biesinger MC, Payne BP, Grosvenor AP, Lau LW, Gerson AR, Smart RSC. Resolving surface chemical states in XPS analysis of first row transition metals, oxides and hydroxides: Cr, Mn, Fe, Co and Ni. *Applied Surface Science* **257**, 2717-2730 (2011).

6. Scott SB*, et al.* The low overpotential regime of acidic water oxidation part II: trends in metal and oxygen stability numbers. *Energy & Environmental Science* **15**, 1988-2001 (2022).

7. Fierro S, Nagel T, Baltruschat H, Comninellis C. Investigation of the oxygen evolution reaction on Ti/IrO2 electrodes using isotope labelling and on-line mass spectrometry. *Electrochemistry Communications* **9**, 1969-1974 (2007).
